# Winter-dormant shoot apical meristem in poplar trees shows environmental epigenetic memory

**DOI:** 10.1093/jxb/ery271

**Published:** 2018-08-09

**Authors:** Anne-Laure Le Gac, Clément Lafon-Placette, Didier Chauveau, Vincent Segura, Alain Delaunay, Régis Fichot, Nicolas Marron, Isabelle Le Jan, Alain Berthelot, Guillaume Bodineau, Jean-Charles Bastien, Franck Brignolas, Stéphane Maury

**Affiliations:** 1 LBLGC, INRA, Université d’Orléans, Orléans, France; 2 MAPMO, CNRS, Université d’Orléans, UMR, Orléans, France; 3 AGPF, INRA, UMR, Ardon, France; 4 Silva, INRA Grand Est, Nancy, AgroParisTech, Université de Lorraine, UMR, Nancy, France; 5 FCBA Délégation Territoriale Nord-Est, Charrey-Sur-Saône, France; 6 GBFOR, INRA, UE, Ardon, France

**Keywords:** Differentially methylated regions, DNA methylation, drought, environment, epigenetics, field grown, poplar, shoot apical meristem, stress memory

## Abstract

Trees have a long lifespan and must continually adapt to environmental pressures, notably in the context of climate change. Epigenetic mechanisms are doubtless involved in phenotypic plasticity and in stress memory; however, little evidence of the role of epigenetic processes is available for trees growing in fields. Here, we analyzed the possible involvement of epigenetic mechanisms in the winter-dormant shoot apical meristem of *Populus* × *euramericana* clones in memory of the growing conditions faced during the vegetative period. We aimed to estimate the range of genetic and environmentally induced variations in global DNA methylation and to evaluate their correlation with changes in biomass production, identify differentially methylated regions (DMRs), and characterize common DMRs between experiments. We showed that the variations in global DNA methylation between conditions were genotype dependent and correlated with biomass production capacity. Microarray chip analysis allowed detection of DMRs 6 months after the stressful summer period. The 161 DMRs identified as common to three independent experiments most notably targeted abiotic stress and developmental response genes. Results are consistent with a winter-dormant shoot apical meristem epigenetic memory of stressful environmental conditions that occurred during the preceding summer period. This memory may facilitate tree acclimation.

## Teaser

The winter-dormant shoot apical meristem of the tree species *Populus* keeps an epigenetic memory of environmental variations that arose during the preceding vegetative period.

## Introduction

Trees are sessile perennial organisms, which therefore respond continuously to environmental pressures over many years. Understanding the mechanisms underlying phenotypic plasticity and stress memory in trees is of utmost importance in the context of rapid climate change (Gagliano *et al.*, 2014). Phenotypic plasticity is the ability of a genotype to rapidly generate and display different phenotypes under distinct environmental conditions (Sultan, 2000; Nicotra *et al.*, 2010). While several studies have been conducted for annual plants (Bossdorf *et al.*, 2010; Kooke *et al.*, 2015; Hébrard *et al.*, 2016), DNA methylation under stress conditions has been studied for only a limited number of trees at the genome level to investigate its putative role in phenotypic plasticity and ecophysiological traits (Conde *et al.*, 2017; Lafon-Placette *et al.*, 2018). Indeed, chromatin marks such as DNA methylation (Niederhuth and Schmitz, 2017) provide strong plasticity and modulate the development, morphology, and physiology of plants by controlling gene expression and the mobility of transposable elements (TEs) (Jablonka *et al.*, 1995; Pál and Miklós, 1999; Angers *et al.*, 2010; Mirouze and Paszkowski, 2011; Kooke *et al.*, 2015; Ong-Abdullah *et al.*, 2015; Yakovlev *et al.*, 2016; Richards *et al.*, 2017).

Recent studies have highlighted how DNA methylation helps trees adapt to changing environments and favors the functional diversity associated with productivity and population stability (Choi and Sano, 2007; Bräutigam *et al.*, 2013; Latzel *et al.*, 2013; Rico *et al.*, 2014; Kawakatsu *et al.*, 2016; Richards *et al.*, 2017). Methylation-induced modifications may be either reversible or retained during cell division (an epigenetic phenomenon) and passed on to daughter cells, as occurs during vernalization (intragenerational transmission) or to the next generation, as shown in EpiRIL (EPIgenetic Recombinant Inbred Lines) populations for inter-generational transmission (Johannes *et al.*, 2009; Reinders *et al.*, 2009; Baulcombe and Dean, 2014; Kooke *et al.*, 2015; Meyer, 2015; Kawakatsu *et al.*, 2016). Another well-described footprint or ‘memory’ system in plants is defense priming, which controls responses to pathogen or herbivore attacks (Pastor *et al.*, 2013; Espinas *et al.*, 2016; Mauch-Mani *et al.*, 2017). The concept of priming has also been applied to responses to abiotic stresses, such as water deficit (Sultan *et al.*, 2009; Ding *et al.*, 2012; Fleta-Soriano and Munné-Bosch, 2016; Mauch-Mani *et al.*, 2017). The priming event is followed by a period of stress memory, which involves storing information about the priming stress, potentially through an epigenetic phenomenon, and results in a modified response upon recurring exposure to a stress or a sustained response after the priming stress (Lämke and Bäurle, 2017). This memory may last from several days to weeks for somatic stress memory, and in some cases may even extend to offspring (Lämke and Bäurle, 2017). For instance, the response to repeated drought stress in *Arabidopsis thaliana* is mediated by transcriptional memory; that is, an increase in the transcription levels of stress response genes occurs (Ding *et al.*, 2012; Avramova, 2015). Hyperosmotic stress memory in *A. thaliana* is also associated with distinct regions of the Arabidopsis genome that are susceptible to DNA (de)methylation. Furthermore, this memory is transmitted to the immediate progeny through the female lineage, due to widespread DNA glycosylase activity in the male germline (Wibowo *et al.*, 2016). Interestingly, genes related to stress memory have been found to be partially conserved between *A. thaliana* and *Zea may*s when the plants are exposed to the same constraints, suggesting that memory could be an evolutionarily conserved response to repeated abiotic stress (Ding *et al.*, 2014). This memory is particularly crucial for perennial organisms such as trees, since episodes of drought are predicted to increase in both frequency and intensity with ongoing global climate change (Allen *et al.*, 2010; IPCC, 2014). Evidence of an epigenetic memory in trees has been described in Norway spruce: different temperatures during embryogenesis induced changes in adult tree traits such as bud phenology or frost tolerance, suggesting an epigenetic memory transmitted from the embryo to the adult plant (Yakovlev *et al.*, 2012). This temperature-related epigenetic memory modifies the expression of bud burst-related genes in epitypes and affects the timing of bud burst in the progeny (Carneros *et al.*, 2017).

Poplar (*Populus* spp.) is a model tree owing to its widely available genome, the large number of genetic and phenotypic variations it exhibits, and its fast growth associated with large water requirements. This makes poplar ideal for dissecting the relationship between the ecophysiological and molecular determinants of water deficit tolerance (Tuskan *et al.*, 2006; Jansson and Douglas, 2007). In this context, a better characterization of the epigenetic component of tree phenotypic plasticity and stress memory seems crucial (Bräutigam *et al.*, 2013; Plomion *et al.*, 2016). Previous studies on poplar have shown that global DNA methylation varies across hybrids and is correlated with biomass productivity and water deficit (Gourcilleau *et al.*, 2010; Raj *et al.*, 2011). Site-dependent hemimethylation statuses and potential responses to environmental and edaphic conditions have also been reported (Guarino *et al.*, 2015). In addition, gene-body DNA methylation in poplar, as compared with Arabidopsis, is more extensive in the open chromatin state; it is linked to structural gene characteristics and is correlated with tissue-specific gene expression or stress (Vining *et al.*, 2012; Bräutigam *et al.*, 2013; Lafon-Placette *et al.*, 2013; Liang *et al.*, 2014; Lafon-Placette *et al.*, 2018). The importance of poplar’s epigenetic component has recently been underlined in site-dependent growth performance (Schönberger *et al.*, 2016) and in the developmental phenotypic plasticity of shoot apical meristem (SAM) in response to environmental bud-break conditions (Conde *et al.*, 2017) and water availability (Lafon-Placette *et al.*, 2018).

**Fig. 1.**
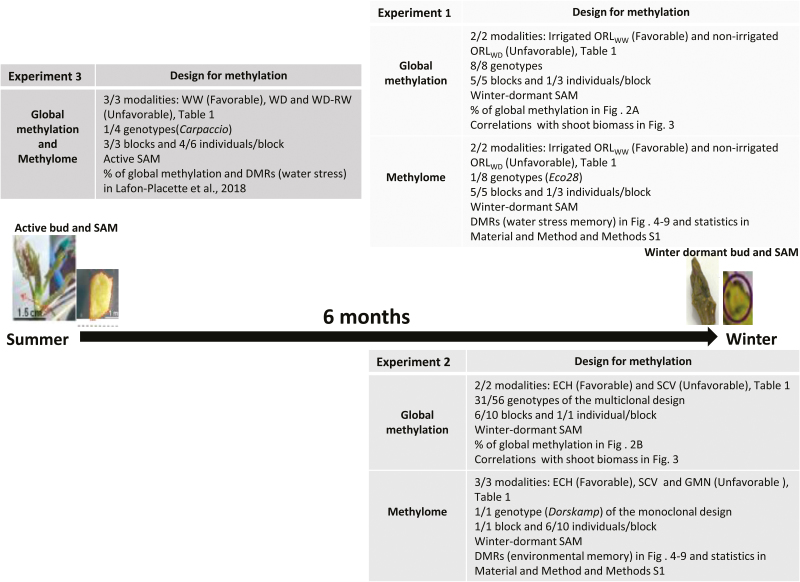
Summary of the three experiments used for the methylome comparative approach. Plant materials, water regimes, samples, and the previously published ecophysiological or epigenomic data used for the analyses in this paper are cited. For experiment 1, the field trial was located near Orléans, France, under two different water regimes (ORL_WW_ and ORL_WD_); WW, Well watered (considered favorable for growth); WD, water deficit (considered unfavorable for growth). For experiment 2, field trials were located in Echigey (a site with favorable growing conditions) and Saint-Cyr-en-Val or Guémené, France (both sites with unfavorable growing conditions). For experiment 3, poplar trees were grown in a greenhouse under three water regimes: WW, WD, and water deficit followed by rewatering (WD-RW), also considered unfavorable for growth. SAM, Shoot apical meristem.

In the present study, we provide new insights into the epigenetic memory of environmental stress in poplar SAM. We analyzed global variations in DNA methylation levels in winter-dormant SAM of *Populus deltoides* × *Populus nigra* genotypes that had faced contrasted growing conditions the previous summer, making use of both a field controlled-drought trial (Experiment 1; Fichot *et al.*, 2010, 2011; Table 1, Fig. 1) and pedoclimatic gradient among three sites in France (Experiment 2; Toillon *et al.*, 2013*a*, b; Table 1, Fig. 1). For global DNA methylation, we used high-performance liquid chromatography (HPLC) to investigate variations in DNA methylation (see Causevic *et al.*, 2005; Gourcilleau *et al.*, 2010; Trap-Gentil *et al.*, 2011; Zhu *et al.*, 2013) and the methylated DNA immunoprecipitation microarray (MeDIP-chip) approach to analyze epigenomic profiles (Hébrard *et al.*, 2016; Lafon-Placette *et al.*, 2018).

**Table 1.** Description of the three experiments used for the comparative approach

		Experiment 1	Experiment 2		Experiment 3
**Environmental conditions**	**Type**	Outdoor plantations	Outdoor plantations		Greenhouse
	**Location**	Orléans (ORL), France	Echigey (ECH) and Saint-Cyr-en-Val (SCV), France	Echigey (ECH), Saint- Cyr-en-Val (SCV), and Guémené (GMN), France	Nancy, France
	**Treatments studied**	Water availability	Pedoclimatic conditions (mainly water availability and soil fertility)	Pedoclimatic conditions (mainly water availability and soil fertility)	Water availability
	**Number of treatment conditions**	2	2	3	3
	**Treatment conditions**	Irrigated (ORL_WW_, favorable) versus non-irrigated (ORL_WD_, unfavorable)	ECH (favorable) versus SCV (unfavorable)	ECH (favorable) versus SCV or GMN (unfavorable)	Well-watered (WW, favorable) versus water deficit (WD, unfavorable) or rewatered (WD- RW, unfavorable)
	**General characterization**	ORL_WW_: Pre‐dawn leaf water potential above –0.20 MPa	ECH: Summer soil VWC >40% (fertile site)	ECH: Summer soil VWC >40% (fertile site)	WW: Soil REW maintained close to 100%
		ORL_WD_: Pre‐dawn leaf water potential of –0.75 MPa at the drought peak	SCV: Summer soil VWC <20% (poor site)	SCV: Summer soil VWC <20% (poor site)	WD: Soil REW maintained at 20% during 2 weeks
				GMN: Summer soil VWC <20% (poor site)	WD-RW: Soil REW maintained at 20% during 8 days followed by rewatering at field capacity for 6 days
**Plantation design**	**Culture**	Coppice	Coppice	Coppice	Potted cuttings
	**Species**	*P. deltoides* × *P. nigra*	*P. deltoides* × *P. nigra*		*P. deltoides* × *P. nigra* and *P. trichocarpa*
	**Number of genotypes**	8	56	1	4
	**Design**	Randomized multiclonal blocks	Randomized multiclonal blocks	Monoclonal blocks	Randomized multiclonal blocks
	**Number of blocks**	5	10	1	3
	**Number of individuals per genotype per block**	3	1	10	6
	**Plantation year**	2006	2009 (ECH), 2010 (SCV)	2009 (ECH, GMN), 2010 (SCV)	2008
	**Coppice year**	2007, 2008	2010 (ECH), 2011 (SCV)	2010 (ECH, GMN), 2011 (SCV)	
	**Sampling year**	2008	2010 (ECH), 2011 (SCV)	2010 (ECH, GMN), 2011 (SCV)	2008
	**Age at sampling time**	3 years	2 years	2 years	3 months
**Previously highlighted effects**	**Main shoot annual dry mass**	G×E ns, G*, E**	G×E***, G***, E***		
	**Relative growth rate in height**	G×E ***, G*, E**			G×E ns, G***, E***
	**References**	Fichot *et al.*, 2010, 2011	Toillon *et al.*, 2013*a*	Toillon *et al.*, 2013*b*	Bizet *et al.*, 2015; Lafon- Placette *et al.*, 2018

REW, Relative extractible water; SAM, shoot apical meristem; VWC, volumetric water content. Effect of growing environments was evaluated with an ANOVA test (G, genotype effect; E, environment effect for growth conditions; G×E, interaction between genotype and environment). Levels of significance: *P<0.05, **P<0.01, ***P<0.001; ns, non-significant. The corresponding references for these data are indicated.

The main aims of the study were (i) to assess the range of genetic and environmentally induced variations in global DNA methylation in winter-dormant SAMs and evaluate the relationship with biomass production, (ii) to identify differentially methylated regions (DMRs) in winter-dormant SAMs of trees that had been exposed to different environmental conditions during their vegetative period for each experiment separately, and (iii) to identify conserved DMRs between the two experiments and the corresponding overlapped genes potentially serving as a signature of water stress or unfavorable growth conditions. Overall, we found that global DNA methylation in winter-dormant SAMs varied in response to environmental conditions and among genotypes, and was correlated with biomass production capacity. Winter-dormant SAMs of trees grown in different conditions during the vegetative period exhibited DMRs between favorable and unfavorable vegetative growth periods inside independent experiments, consistent with an epigenetic memory of environmental conditions mediated by DNA methylation that lasted for at least 6 months. In our study, epigenetic memory corresponded to the DMRs that were stable through mitosis in SAM from the time when the induction was performed (summer, active SAM) to the time when we performed our analyses (winter, dormant SAM). In addition, we compared the DMRs reported here with DMRs found for active SAMs in greenhouse-grown trees (Lafon-Placette *et al.*, 2018; Table 1, Fig. 1). We detected conserved DMRs among these three independent experiments that mostly overlapped genes related to abiotic stress or developmental responses. Altogether, our data clearly point to an environmentally induced epigenetic memory in the SAM in field-grown poplar trees.

## Materials and methods

### Plant materials, experimental designs, growth performance, and samplings

Analyses were performed on samples collected in three independent experiments conducted in different environments with distinct genotypes. These experiments had initially been set up to characterize the ecophysiological response to water availability and pedoclimatic conditions at the genotype level. A comparative analysis was therefore carried out by merging the ecophysiological data already published (Table 1; Fichot *et al.*, 2010, 2011; Toillon *et al.*, 2013*a*, b; Bizet *et al.*, 2015; Lafon-Placette *et al.*, 2018) and the epigenomic analyses performed in the framework of the present study (Fig. 1).

Experiment 1 consisted of a field drought experiment conducted in a common garden. The experiment was installed in spring 2006 at the INRA Research Station of Forest Genetics in Orléans in central France (Loiret, 47°46ʹN, 1°52ʹE, 110 m above sea level) on poor loamy sand. Hardwood cuttings (25 cm in length) of eight unrelated hybrid genotypes of *P. deltoides* × *P. nigra* were planted in the field (Table 1; Fichot *et al.*, 2010, 2011). The eight genotypes were initially selected for their contrasting growth performance and water-use efficiency (Monclus *et al.*, 2005; Fichot *et al.*, 2009). Two twin plots were established 15 m apart on a 250 m^2^ surface area. Each plot was divided into five complete randomized blocks with three ramets of each genotype per block. All the plants were cut back at the end of 2006 and 2007, thereby creating a coppice system the following years. All the plants were irrigated during the 2007 growing season to ensure successful installation. In 2008, irrigation was maintained on one plot (control plot, ORL_WW_) and withheld from the other plot (water deficit plot, ORL_WD_) from June 18 to the end of September. The unirrigated plot received water only from rainfall. There was a summer drought period peaking on July 24, which resulted in a 25% average decrease in annual above-ground biomass production (Fichot *et al.*, 2010). Additional information on the experimental design, growth conditions, and genotype ecophysiological characteristics can be found in Fichot *et al.* (2010, 2011).

Experiment 2 consisted of a network of site-specific field trials initially established to evaluate genotype × environment interactions in poplars cultivated under short-rotation coppice. Multiclonal field trials were composed of 56 unrelated *P. deltoides* × *P. nigra* genotypes and were replicated on two sites in France: at Echigey (ECH; Côte d’Or, 47°10ʹN 5°11ʹE, 197 m above sea level) and Saint-Cyr-en-Val (SCV; Loiret, 49°81ʹN 1°98ʹE, 100 m above sea level) (Toillon *et al.*, 2013*a*; Table 1; see also Supplementary Table S1 at *JXB* online). Each field trial was composed of 10 complete randomized blocks with one ramet of each genotype per block. Monoclonal trials of the same genotype (*P. deltoides* × *P. nigra* cv. Dorskamp) were also established at the same two sites (ECH and SCV) plus one additional third site: Guémené-Penfao (GMN; Loire-Atlantique, 47°37ʹN 1°49ʹW, 43 m above sea level). The sites were planted in spring 2009 at ECH and GMN, and 2010 at SCV, with 25 cm long woody-stemmed cuttings. Each plantation was managed as an extensive short-rotation coppice using a double-row planting scheme with alternating distances of 0.75 m and 2 m between the rows and 1 m between trees within the rows (~7272 trees ha^−1^). The ECH site was characterized by fertile, silty-clay soil and high soil water content during the growing season (>40% soil volumetric water content at 20 cm) (Toillon *et al.*, 2013*a*). The SCV site was characterized by a poor, sandy-loam soil type with low soil water content (<20%). The GMN site was located on a silty-clay soil with a low soil water content (<20%) (Toillon *et al.*, 2013*b*). ECH was the most productive site, followed by SCV and then GMN, which had, respectively, 32.5% and 50% less annual average above-ground biomass production than ECH (Toillon *et al.*, 2013*a*, b). Growth conditions were considered ‘favorable’ at the ECH site and ‘unfavorable’ at both the GMN and SCV sites. Additional information on the experimental design, growth conditions, and genotype ecophysiological characterization can be found in Toillon *et al.* (2013*a*, b).

Experiment 3 consisted of plants of four commercial hybrids, *P. deltoides* × *P. trichocarpa* (cv. Beaupré) and *P. deltoides* × *P. nigra* (cv. Carpaccio, Dorskamp, and Soligo), grown in 10 liter pots in Nancy, France in April 2008 (Table 1; Bizet *et al.*, 2015, Lafon-Placette *et al.*, 2018). The plants were exposed to natural daylight (400–900 μmol m^−2^ s^−1^). Air temperature and humidity were maintained within the ranges of 19–26 °C and 50–75%, respectively. After 5 weeks at field capacity, the plants were organized in three randomized blocks with six ramets for each genotype per block and then exposed to three distinct water treatments: (i) well watered for 10 days, where evaporative demand was compensated for by watering to field capacity, (ii) water deficit for 10 days, where the relative extractable water content was maintained within the range of 17–23%, or (iii) rewatering, where water deficit was applied for 8 days, followed by rewatering to field capacity for 6 days. Additional information on the experimental design, growth conditions, and genotype ecophysiological characterization can be found in Bizet *et al.* (2015) and Lafon-Placette *et al.* (2018).

Winter-dormant buds from *P. deltoides* × *P. nigra* genotypes were collected for epigenomic analysis from experimental systems 1 and 2 (Fig. 1). For experiment 1, dormant SAMs were collected for all eight genotypes in winter 2008, 6 months after the summer drought (Table 1, Fig. 1). For experiment 2, dormant SAMs were collected in winter 2010 (at the ECH and GMN sites) and winter 2011 (at the SCV site) 6 months after the summer period for both the multiclonal and monoclonal trials (Table 1, Fig. 1). For the multiclonal trials, SAMs were picked from a subset of 31 genotypes representative of the genetic diversity among the 56 genotypes available. Next, the shoot apices were cut in half with a scalpel and all visible differentiated tissues were discarded under a binocular magnifying glass in order to extract, as much as possible, the SAM, as previously reported (Lafon-Placette *et al.*, 2013). Samples were frozen in liquid nitrogen and stored at –80 °C.

For methylome analysis, we selected for experiment 1 the winter-dormant SAM of the Eco 28 genotype, which showed the highest relative growth rate for the main shoot under well-watered conditions and the most significant effect of drought on DNA methylation (Fichot *et al.*, 2010; Fig. 1); for experiment 2 the winter-dormant SAM of the Dorskamp genotype owing to its positive and well-reported physiological response to moderate drought (Marron *et al.*, 2002, 2003; Toillon *et al.*, 2013*a*; Fig. 1); and in experiment 3 the active SAM of the Carpaccio genotype owing to its positive and well-reported physiological response to moderate drought (Fig. 1). For global DNA methylation, methylome, and transcriptomic analyses, the corresponding data have been reported in Lafon-Placette *et al.* (2018).

### DNA extraction and determination of global DNA methylation percentage by HPLC

Following Lafon-Placette *et al.* (2013, 2018), the winter-dormant SAMs from experiments 1 (eight genotypes) and 2 (31 genotypes) were cleared of all visible differentiated tissues to maximize the isolation of the meristematic cells, immersed in liquid nitrogen, and ground to a fine powder in an automatic ball mill (MM 200 Retsch, Germany). Genomic DNA was extracted following the CTAB protocol (Doyle and Doyle, 1987; Lafon-Placette *et al.*, 2018) and stored at –80 °C. The quantity and quality of DNA were estimated with a NanoDrop spectrometer (NanoDrop Instrument, France).

Genomic DNA was enzymatically hydrolyzed into nucleosides and analyzed by HPLC with a GeminiTM column (150 × 4.6 mm, 5 µm; Phenomenex, Le Pecq, France) and an isocratic mobile phase consisting of 0.5% methanol (v/v) and 5 mM acetic acid in water, as described by Zhu *et al.* (2013). Controls for this procedure included co-migration with commercial standards (Sigma-Aldrich), confirmation by enzyme restriction analysis, and tests for RNA contamination based on the HPLC detection of ribonucleosides. Global DNA methylcytosine percentages (% mC) were calculated as follows:

%mC=(mC/(C+mC))×100,

where C is the 2ʹ-deoxycytidine content and mC is the 5-methyl-2ʹ-deoxycytidine content. For each set of conditions, we analyzed SAMs from four to six individuals per genotype (depending on the possible degradation of SAM for some individuals in the field), with two hydrolysis replicates and two HPLC runs for each.

### Methyl DNA immunoprecipitation and microarray analysis

Custom microarray probes designed with eArray software (https://earray.chem.agilent.com/earray/; Agilent Technologies, Massy, France) using *Populus trichocarpa* genome v2 (Tuskan *et al.*, 2006; http://www.phytozome.net/poplar.php) have been described by Lafon-Placette *et al.* (2018) (GEO accession number GSE46624). For each poplar gene model (over 42000 models), we designed five probes: one probe bound to the 0.5 kb upstream from the start codon (annotated ‘PROMOTER’), one probe bound to the 0.5 kb downstream from the stop codon, and three probes bound within the body of the gene (located between the transcription start site and transcription termination site, and annotated ‘BODY’,). Probes were annotated ‘TE’ if they were identified in a transposable element (TE) described by the RepeatMasker annotation of the *P. trichocarpa* v3 genome (http://www.phytozome.net/poplar.php). If the probe was present in either a TE inserted into a gene or in a promoter, it was annotated as ‘BODY+TE’ or ‘PROM+TE’, respectively. Otherwise, the probe was annotated as ‘INTERGENIC’. Additional probes covering genes and intergenic regions were also selected based on a previous study (Lafon-Placette *et al.*, 2013). These probes spanned from 2 kb upstream to 2 kb downstream from target loci (either genes or intergenic regions), with a distance between probes of 140 bp for genes and 780 bp for intergenic regions. The microarray also contained 50 probes for reproducibility controls and internal Agilent control probes. Following the release of the v3 *P. trichocarpa* genome sequence, probe information was updated by BLAST searches of probe sequences against the v3 genome sequence. See Hébrard *et al.* (2016) and Lafon-Placette *et al.* (2018) for a description of the procedure for MeDIP-chip, including controls and data analysis under the supervision of IMAXIO (Clermont-Ferrand, France), in accordance with the instructions issued by Agilent Technologies.

For experiments 1 and 2, the set of winter-dormant SAM samples used for methyl DNA immunoprecipitation was composed of four to six individuals for one genotype (Eco28 for experiment 1 and Dorskamp for experiment 2) for each condition (Fig. 1) depending on the possible degradation of the SAM for some individuals in the field.

### DMR identification and bioinformatics analyses

DNA methylation data obtained with the MeDIP approach were used to detect DMRs between favorable and unfavorable conditions for each experiment. For experiment 1, we compared the Eco 28 genotype in favorable well-watered (ORL_WW_) and unfavorable water-deficit (ORL_WD_) conditions. For experiment 2, we compared the Dorskamp genotype in favorable growing conditions at the ECH site and in unfavorable conditions at SCV or GMN. Identifying DMRs within each experiment allowed us to compare favorable and unfavorable conditions for each experiment without taking into account differences among experiments for the growing design.

To identify DMRs, we applied a mixed model to normalized and filtered signal probes mapped on to the poplar reference genome (ftp://plantgenie.org/Data/PopGenIE/Populus_trichocarpa/v3.0) and estimated the false discovery rate (FDR) for each set of biological conditions, following Lafon-Placette *et al.* (2018) (see Supplementary Method S1). Briefly, we first estimated the reference mean from 20% of the probes selected at random, for each chromosome under each growth condition and for each experiment. This became our reference mean, or base level of methylation, for a given chromosome in a given biological sample. We used mixed models based on the bimodal empirical distributions for this estimated reference mean (McLachlan and Peel, 2000). We used the R statistical software mixtool package (Benaglia *et al.*, 2009) for subsequent analyses. We first constructed 50 kb analysis windows (~7550 windows) based on the remaining 80% of the probes, following Lafon-Placette *et al.* (2018). Each defined window was characterized by its specific probe composition and gene model positions. These windows were used to assess whether the mean methylation signal per window differed from the previously determined reference mean. Of the windows, 56% had fewer than 30 probes for experiments 1 and 71% had fewer than 30 probes for experiment 2. We therefore performed a one-sample Wilcoxon signed-rank non-parametric test with a symmetric distribution of the data around the reference mean as the null hypothesis. This test was performed to compare each window mean with the corresponding reference mean to obtain a *P*-value that would allow us to accept or reject the null hypothesis. An FDR was then used to control for multiple testing (Benjamini and Hochberg, 1995) with α=5%, to control for the global risk of error in the windows selected by the multiple Wilcoxon signed-rank tests. This FDR was estimated with the R package fdrtool (Strimmer, 2008). The results were ranked according to decision rule scores based on the FDR. We defined three levels: not significantly different from the mean reference level of methylation (0), significantly lower levels of methylation than the reference mean (–1), and significantly higher levels of methylation than the reference mean (+1). DMR was defined as a window for which different decision rule scores were obtained for favorable and unfavorable growth conditions for each experiment (0 and +1, 0 and –1, or +1 and –1).

To assess Gene Ontology (GO) term enrichment for the genes inside DMRs, we used the corresponding best BLAST hit for each v3.0 poplar model gene from the best v3.0 BLAST hits with Arabidopsis TAIR10. AgriGO (http://bioinfo.cau.edu.cn/agriGO/) and then REVIGO (http://revigo.irb.hr/) software, set to default parameters, were used for enrichment analysis. A treemap was created with rectangle size adjusted to reflect the absolute log_10_*P*-value of the GO term.

### Statistical analyses

Statistical analyses were performed with Rstudio statistical software (R Core Team, 2015; http://www.rstudio.com/). Means are expressed with their standard error. Differences between groups were evaluated by ANOVA (one-way ANOVA test, general linear model procedure, two-way ANOVA test, genotype and treatment effect). Tukey rank tests were also performed *post hoc*, considering only the effect of genotype in a given set of conditions. Relationships between global DNA methylation and ecophysiological parameters were analyzed for normality by carrying out Shapiro–Wilk tests. Linear correlation analyses were then carried out to determine Pearson’s coefficient (*r*) and Spearman’s coefficient (rho). Significant effects of the methylation pattern on the distribution of DMRs were evaluated with the chi-squared (χ^2^) test. Finally, a hypergeometric distribution was used to describe the probability of having more common DMRs than the number identified without replacement. The *n* value indicated in the figure legends corresponds for the ANOVA test and the Pearson correlation to the lowest number of biological replicates for a given condition in an experiment (Fig. 1). Some SAMs could not be harvested owing to the field conditions (e.g. insect or pathogen attack).

### Accession numbers

Microarray design and methylome data are available from the GEO database (accession numbers GSE46624 and GSE97144, respectively).

## Results

### Global DNA methylation in winter-dormant SAMs depends on genotype and environment and correlates with biomass production

Global DNA methylation was measured by HPLC on winter-dormant SAMs of the different poplar genotypes from the two independent experiments 1 and 2, comparing in each case favorable and unfavorable growing conditions (Fig. 1, Table 1).

**Fig. 2.**
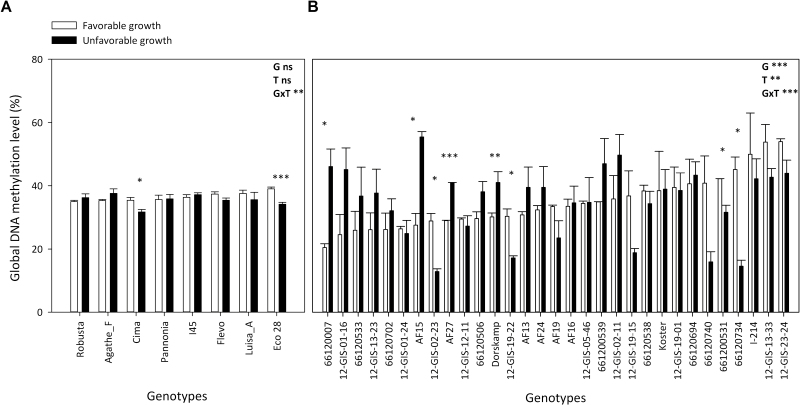
Genotypic variation and environmental effect for global DNA methylation on different *Populus deltoides* × *P. nigra* hybrids grown in experiment 1 (A) or experiment 2 (B). White and black bars correspond respectively to well-watered (ORL_WW_, favorable) and water deficit (ORL_WD_, unfavorable) conditions for experiment 1 (Fichot *et al.*, 2010, 2011), and to favorable (ECH) and unfavorable (SCV) growth conditions for experiment 2 (Toillon *et al.*, 2013*a*). Values are genotype means ±SE (*n*=4). Global genotype variations and the effect of growing conditions were evaluated by ANOVA. G, genotype effect; T, treatment effect for growth conditions; G×T: interaction between genotype and treatment. *P<0.05, **P<0.01, ***P<0.001; ns, non-significant.

In experiment 1, global DNA methylation varied between 34% and 39% across the eight unrelated poplar genotypes (Agathe_F, Cima, Eco28, Flevo, I45-51, Luisa_Avanzo, Pannonia, and Robusta) and the two water regimes (ORL_WW_ versus ORL_WD_; Fig. 2A). A significant genotype×treatment interaction was detected; two genotypes (Cima and Eco 28) displayed significant differences between well-watered and water deficit conditions, and in both cases a decrease in the level of DNA methylation was observed in water deficit conditions (Fig. 2A). These two genotypes are among the three most productive of the eight analyzed genotypes in terms of relative growth rates of the main shoot under well-watered conditions and exhibiting a significant decrease of their relative growth rate in response to water deficit (Fichot *et al.*, 2010).

A larger range of variation was found in experiment 2, where global DNA methylation varied between 20% and 54% across the 31 unrelated genotypes and the two locations (ECH, favorable, versus. SCV, unfavorable; Fig. 2B). A significant genotype×treatment interaction was detected (Fig. 2B). Significant differences across sites were detected for 8 out of 31 genotypes. Four genotypes displayed higher DNA methylation under favorable conditions, while the other four showed lower DNA methylation under favorable conditions (Fig. 2B). No relationship between genetic proximity and variations in global DNA methylation was found (Supplementary Table S1). For example, clones 12-GIS-13–23 and 12-GIS-13–33 came from the same genetic background but showed opposite patterns of variation in their global DNA methylation levels (Supplementary Table S1; Fig. 2B).

**Fig. 3.**
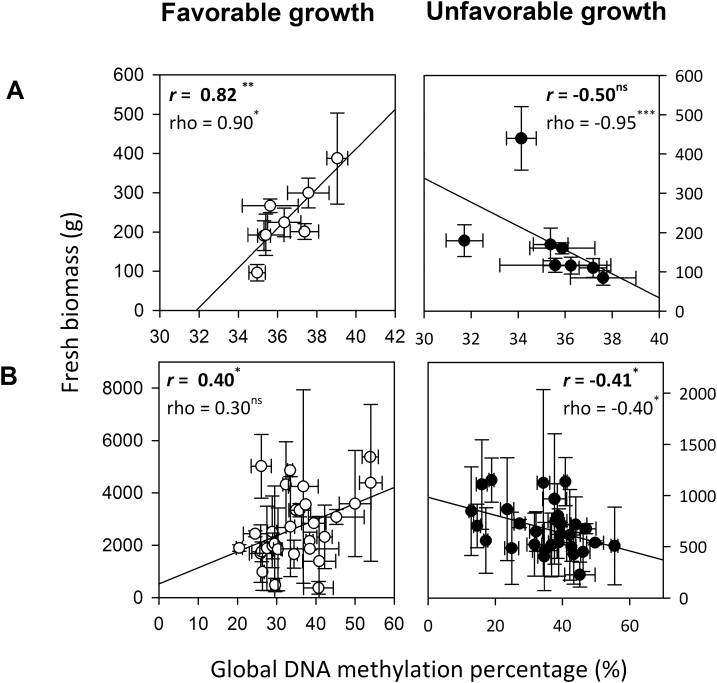
Relationships between global DNA methylation percentage and above-ground fresh shoot biomass for experiment 1 (A) and experiment 2 (B). White circles are for favorable (ORL_WW_, ECH) growing conditions and black circles for unfavorable (ORL_WD,_ SCV) conditions. Values are genotype means (*n*=4) and the line represents the linear regression correlation among individuals. Shapiro tests were used to confirm values given by a homogeneities distribution test. Linear parametric correlation analyses were then carried out to determine Pearson’s coefficient (*r*) and Spearman’s coefficient (rho).

Significant positive correlations were detected between global DNA methylation and shoot biomass under favorable growing conditions for both experiments (Fig. 3), while under unfavorable growing conditions these correlations were negative (Fig. 3).

### Winter-dormant SAM displays hyper- and hypomethylated regions in response to environmental variations from the preceding vegetative period

**Fig. 4.**
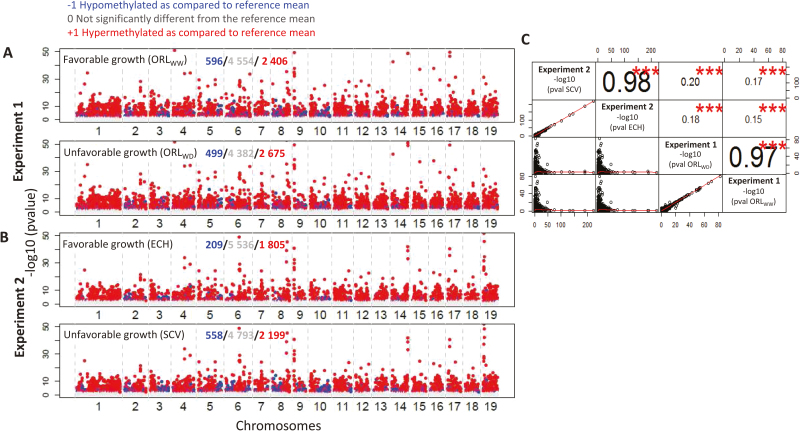
Genomic features of DNA methylation changes in poplar shoot apical meristem in response to environmental variations. (A) Experiment 1: Favorable (ORL_WW_) and unfavorable (ORL_WD_, unfavorable) growing conditions. (B) Experiment 2: Favorable ( ECH) and unfavorable (SCV) growing conditions. Graphs are based on Manhattan plots from a significant false discovery rate of 5%. The plots show –log_10_*P*-values on the y axis, and the location of the different 50 kb windows in the genome with a gap at chromosome locations on the x axis. Blue dots correspond to hypomethylated windows compared with the reference mean, red dots to hypermethylated windows compared with the reference mean, and grey dots to non-significant windows. (C) Relationships (linear correlations) between –log_10_ of experiment 1 and 2 windows for all growth conditions (*n*=7550). Lines represent linear regression correlations; numbers represent Pearson’s coefficient (*r*). *P<0.05, **P<0.01, ***P<0.001; ns, non-significant.

The methylome of winter-dormant SAMs from trees grown in experiments 1 and 2 was assessed by MeDIP-chip analysis on one genotype from each experiment (Fig. 1). *P. deltoides* × *P. nigra* clones of genotype Eco28 were used for experiment 1 and of genotype Dorskamp for experiment 2. A custom-made poplar array (1 million probes) was generated as described by Hébrard *et al.* (2016) and Lafon-Placette *et al.* (2018). In order to identify DMRs, consecutive 50 kb genomic windows (~7550 windows; see Supplementary Method S1) were first calculated within each biological condition, as described by Lafon-Placette *et al.* (2018). Manhattan plots were generated (Figs 4A, B; Supplementary Fig. S1A) to represent DNA methylation variations along the 19 chromosomes as –log_10_*P*-values for all the consecutive 50 kb genomic windows, ranging from 0 to ~50 (Fig. 4A). One dot represents one 50 kb genomic window and DNA methylation status is color-coded as follows: not significantly different from the average level of methylation (designated 0; grey dots in Fig. 4A), significantly lower (hypomethylated, designated −1; blue dots in Fig. 4A), and significantly higher (hypermethylated, designated +1; red dots in Fig. 4A; see Materials and methods and Supplementary Method S1).

**Fig. 5.**
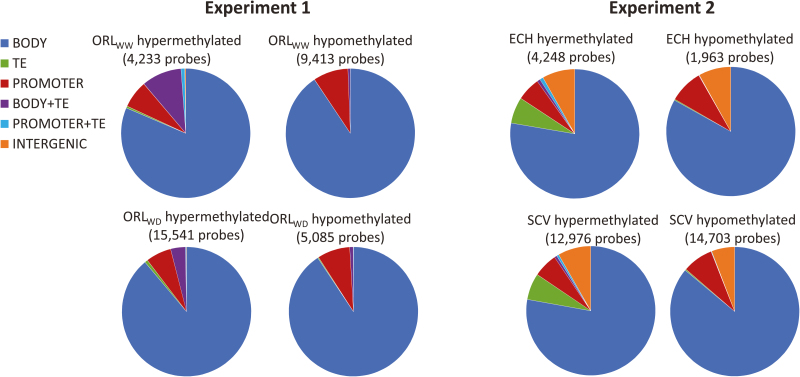
Characterization of loci overlapped by DNA methylation changes (DMRs) in response to different environmental conditions. DMRs were classified as hypo- or hypermethylated for each experimental design. BODY, gene body; PROMOTER, 1 kb upstream region; TE, transposable element; BODY+TE, TE inserted into a gene body; PROM+TE, TE inserted into a promoter; INTERGENIC, any other locus.

The distribution of hypo- and hypermethylation status over the chromosomes differed significantly between experiments (*r* values ranging from 0.15 to 0.20 at *P*<0.001; Fig. 4C). In contrast, within an experiment, similar distributions of DNA methylation on chromosomes were observed between favorable and non-favorable conditions in the 50 kb genomic windows tested, as indicated by *r* values ranging from 0.97 to 0.98 (at *P*<0.001; Fig. 4C). Despite this stability, differences between favorable and unfavorable conditions for a given experiment were detected as changes in the number of hyper -and hypomethylated loci. Indeed, 596 hypomethylated and 2406 hypermethylated loci were identified in the genome of trees growing in the favorable condition of experiment 1, while under unfavorable growing conditions, fewer hypomethylated (499) and more hypermethylated (2675) loci were detected within the analyzed windows (Fig. 4A). In experiment 2, both hypomethylated and hypermethylated loci increased between favorable and unfavorable conditions: from 209 to 558 for hypomethylated loci, and from 1805 to 2199 for hypermethylated loci (Fig. 4B; Supplementary Fig. S1). We also determined the association between DNA methylation status and specific DNA sequences, including genic regions and transposons. Variations in DNA methylation status observed in the tested windows were mostly located in the genic region between the transcription start site and transcription termination site, thereafter defined as the gene body (Fig 5; Supplementary Fig. S1B; Supplementary Table S2). This could explain in part the differences observed between variations of methylation for a given genotype for global DNA methylation performed by HPLC (mostly affected by non-genic loci such as repeated sequences) and the methylome by MeDIP-chip (mostly affected by genes) (Figs 2 and 4).

The identification of hypo- or hypermethylated regions also confirmed that TEs were mainly hypermethylated, while promoters showed similar proportions of hypo- and hypermethylated regions (Fig. 5; Supplementary Fig. S1B; Supplementary Table S2). In each experiment, no impact of the growing conditions was observed on the distribution of methylation status, while TEs and intergenic loci revealed differences between experiments 1 and 2 (Fig. 5 and Supplementary Fig. S1B). Functional annotation of the genes covered by methylation status variation was performed by using GO enrichment analysis. This analysis was based on the annotations for the homologous genes of Arabidopsis in TAIR10 (https://www.arabidopsis.org/). The analysis revealed that these genes were rich in biological processes (Supplementary Table S5; Supplementary Figs S1C and S2). In the ORL_WD_ condition, for example, some of the main categories were associated with genes involved in multicellular organism development, including shoot and meristem development (37%; *P*=2.7 × 10^−53^), the abiotic stress response (25%; *P*=3.9 × 10^−56^), and the negative regulation of biological processes (13%; *P*=8.8 × 10^−29^) (Supplementary Fig. S2, Supplementary Table S5).

### Winter-dormant SAM maintains DMRs from the preceding vegetative period

**Fig. 6.**
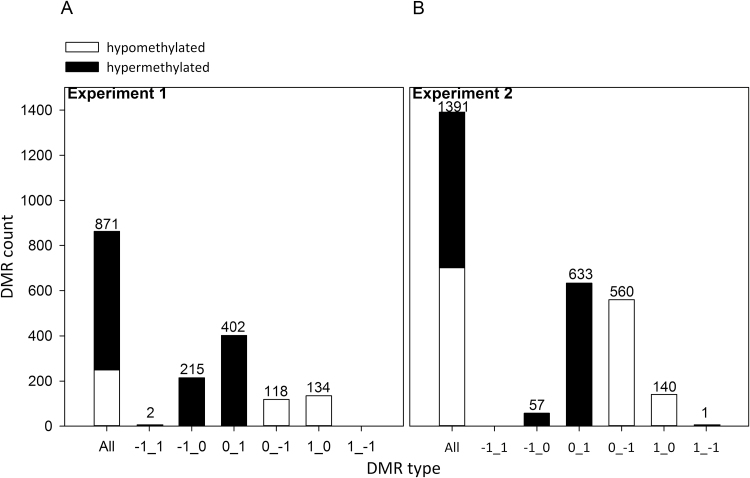
Classification of DNA methylation changes (DMRs) for the intensity of variations for (A) experiment 1 and (B) experiment 2. DMRs were split between hypermethylated (black bars) and hypomethylated (white bars) regions. DMR categories were defined by the size of DNA methylation changes between favorable and unfavorable growing conditions (indicated on the x axis as favorable_unfavorable): mild changes from –1 (hypomethylation) to 0 (average methylation) and from 0 to +1 (hypermethylation), or strong changes from –1 (hypomethylation) to +1 (hypermethylation).

Using the FDR controlling procedure for multiple testing (α=5%; see Supplementary Method S1), 871 DMRs (11.5%) between favorable (ORL_WW_) and unfavorable (ORL_WD_) growing conditions were identified in experiment 1. In experiment 2, 1391 DMRs (18.4%) were detected between favorable (ECH) and unfavorable (SCV) sites (Fig. 6A; Supplementary Table S3). In both experiments, ~70% of the DMRs were hypermethylated under unfavorable compared with favorable conditions (Fig. 6A, B). In all cases, nearly all DMRs showed weak DNA methylation variations (–1 to 0 or 0 to +1 scores; Fig. 6).

GO enrichment analysis was used to characterize the genes associated with DMRs (and by the filtered MeDIP probes) as previously mentioned (Supplementary Figs S3–S5; Supplementary Table S5). Similar functional categories were associated with DMRs in both experiments (Supplementary Fig. S3; Supplementary Table S5), of which the most abundant were cellular protein modification process (with RNA metabolism; 18% of genes, *P*=5.2 × 10^−13^ in experiment 1; Supplementary Table S5; Supplementary Fig. S3A), response to abiotic stress (14% of genes, *P*=5.9 × 10^−14^ in experiment 1), and cellular ketone metabolism (cell cycle, cell division, small molecule metabolism; 10% of genes, *P*=3.1 × 10^−7^ in experiment 1). The GO category corresponding to genes involved in the response to abiotic stress was associated with both hyper- and hypomethylated DMRs (Supplementary Figs S4 and S5). Additional GO categories such as phytohormone signaling/metabolism pathways that were found for jasmonic acid, abscisic acid, and salicylic acid were specifically associated with hypomethylated DMRs, whereas RNA processing and small molecule metabolism were associated with hypermethylated DMRs in experiment 1 (Fig. S4). In experiment 2, a similar observation was made, with GO categories containing salicylic acid metabolism being associated with hypomethylated DMRs and anatomical structure development and macromolecule catabolism categories with hypermethylated DMRs.

In order to determine possible correlations between gene expression and change in methylation levels, we used a candidate approach to characterize genes covered by DMRs using an *in silico* analysis with available transcriptomic data describing differentially expressed genes (DEGs) in the active SAM between the well-watered and the water deficit and rewatering conditions for experiment 3 (Lafon-Placette *et al.*, 2018; Table 1; Fig. 1). This overlap analysis identified 123 genes between DEGs (Lafon-Placette *et al.*, 2018) and DMRs for experiment 1, and 96 genes for experiment 2 (Supplementary Table S4). For experiments 1 and 2, identified genes were enriched in response to stress (29%, *P*=2.3 × 10^−5^ and 33%, *P*=2.2 × 10^−11^, respectively) (Supplementary Table S5; Supplementary Fig. S6). Specific GO categories were also detected, such as hormones (experiment 1: 11%, *P*=5.3 × 10^−4^), regulation of gene expression and epigenetic (experiment 2: 4%, *P*=8.2 × 10^−2^), and shoot development (experiment 2: 3%, *P*=8.2 × 10^−2^) (Supplementary Table S5; Supplementary Fig. S6). The distribution of DEGs in the DMRs was analyzed. The data showed an over-representation, relative to a random distribution, of hypermethylation for down-regulated (*P*<0.01) and up-regulated (*P*<0.001) genes in experiment 1, while hypergeometric tests revealed that DEGs were equally distributed between hypo- and hypermethylated DMRs in experiment 2 (Supplementary Fig. S7).

Finally, we used hypergeometric tests to determine whether the distribution of DEGs over DMRs occurred by chance or not (Supplementary Fig. S7). This revealed that down-regulated DEGs (*P*<0.01) were over-represented in the hypermethylated DMRs found in experiment 1, whereas up-regulated DEGs (*P*<0.001) were equally distributed between hypo- and hypermethylated DMRs in experiment 2.

### SAM displays common environmentally induced epigenetic signatures

**Fig. 7.**
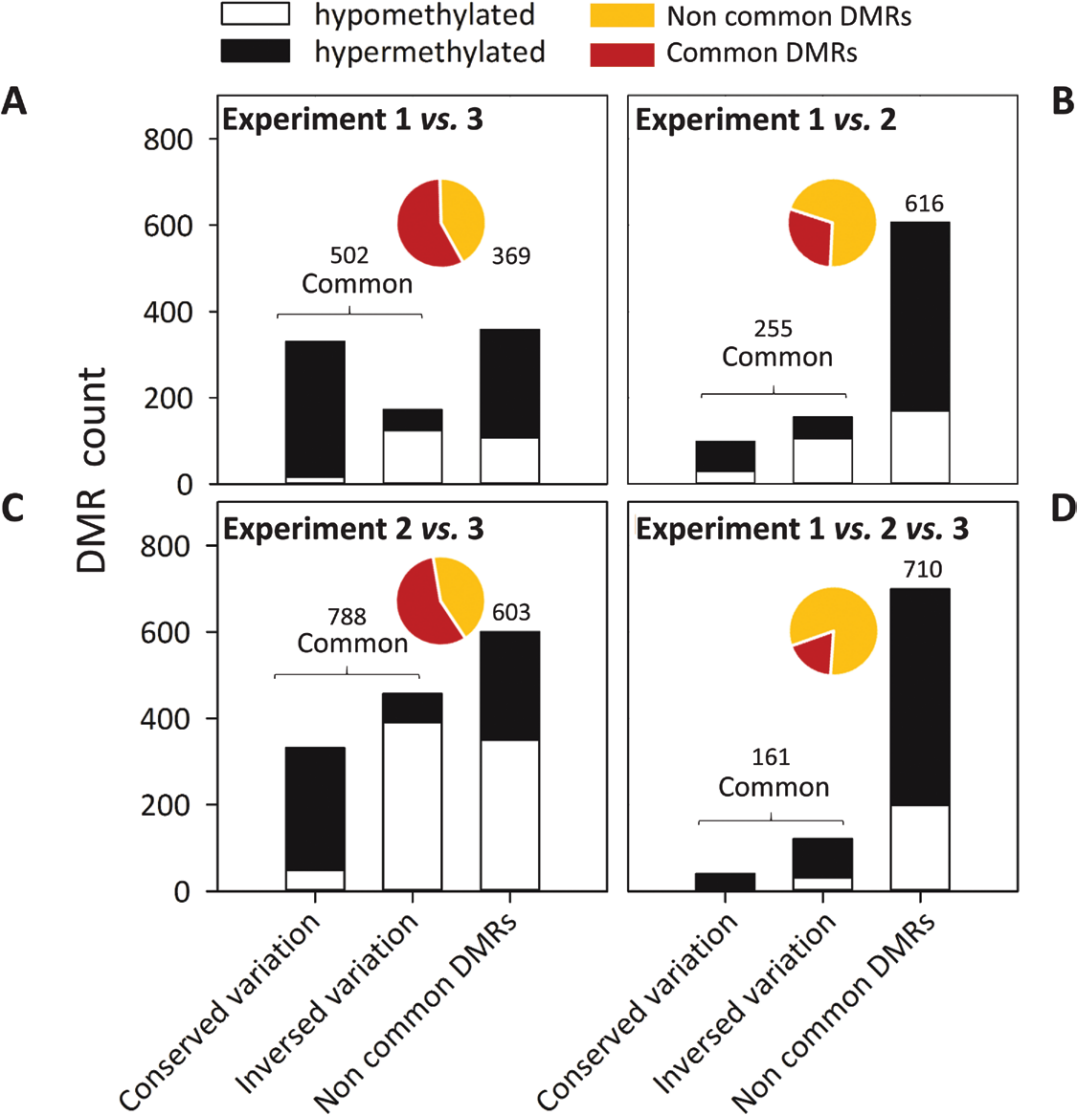
Comparison of DMRs among (A) experiments 1 and 3, (B) experiments 1 and 2, (C) experiments 2 and 3, and (D) experiments 1, 2, and 3. DMRs inside an experiment correspond to a genomic region showing a significant variation in DNA methylation between favorable and unfavorable growing conditions. ‘Common’ DMRs correspond to the same DMRs found in at least two experiments (if not, DMRs are labeled ‘non-common’). For the common DMRs, the direction of methylation variation (hyper- or hypomethylation) between favorable and unfavorable growing conditions can be maintained (conserved DMRs) or reversed (inversed DMRs). Values indicate DMR counts for common (red) and non-common (yellow) categories and are represented as pie charts. Black bars correspond to hypermethylated DMRs and white bars to hypomethylated DMRs.

In order to identify the loci systematically and durably targeted in SAM by DNA methylation variations (DMRs called ‘epigenetic signatures’) depending on site growth performances (favorable versus unfavorable conditions), we compared the DMRs identified in the winter-dormant SAMs of the field experiments 1 and 2 among themselves and with the recently reported DMRs in active SAM of experiment 3 (Lafon-Placette *et al.*, 2018; Figs 1 and 7). More than half of the DMRs identified in winter-dormant SAMs (502 out of 871 DMRs, or 57.6%, in experiment 1; 788 out of 1391 DMRs, or 56.6%, in experiment 2) were common to those found in active SAMs of experiment 3 (Fig. 7A, C). Fewer DMRs (255 out of 871, or 29.2%; Fig. 7B) were common to experiments 1 and 2, and 161 DMRs (18.4 %) were common to all three experiments (Fig. 7D). Using hypergeometric tests on common DMR distribution among the experiments, we identified significant over-representations (*P*<0.001) among all the compared experiments.

Two categories of DMRs common between pairwise comparisons of all the experiments were distinguishable: (i) DMRs with the same direction of methylation changes (i.e. hyper- or hypomethylation) between favorable and unfavorable growth conditions (hereafter called ‘conserved’ DMRs; Fig. 7) and (ii) DMRs with the opposite direction in methylation changes (hereafter called ‘inversed’ DMRs; Fig. 7). Globally, the proportion of common conserved DMRs was lower than of inversed DMRs (Fig. 7B–D). However, notably, experiments 1 and 3 showed more conserved than inversed common DMRs (Fig. 7A). Hypergeometric tests revealed a significant over-representation of hypermethylated DMRs among conserved DMRs relative to a random distribution (*P*<0.001) among all compared experiments (Fig. 7).

**Fig. 8.**
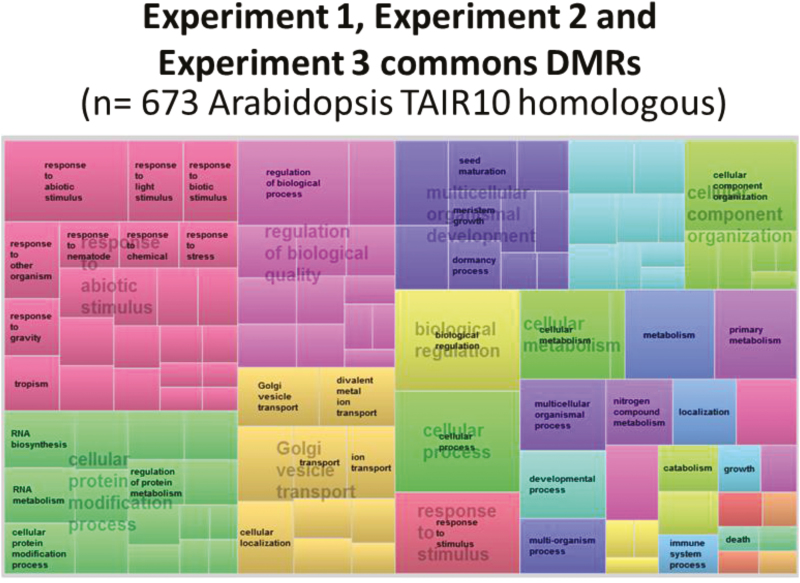
Treemap view of REVIGO for biological process on DMRs common to experiments 1, 2 and 3 (*n*=673 annotations of homologous Arabidopsis genes). Each rectangle is a single cluster representative of TAIR10 corresponding to poplar v3.0 annotations. The representatives are combined into superclusters of loosely related terms, visualized with different colors. Rectangle size is adjusted to reflect the abs log_10_*P*-value of the GO term in the underlying Gene Ontology Annotation database.

GO enrichment analyses were performed on the genes associated with the common DMRs, either globally or separately for the conserved and inversed DMRs (Fig. 8; Supplementary Figs S8–S11; Supplementary Table S5). In all cases, one of the most significant enrichment category corresponded to genes involved in the response to (abiotic) stresses (~15% of the genes; see Supplementary Table S5). GO enrichment also revealed differences between hyper- or hypomethylated DMRs for experiments (1 versus 2; 1 versus 3; 2 versus 3) of common and conserved or inversed DMRs. These data suggest the possibility of coordinated control of different biological functions, for example, by hyper- or hypomethylation. Indeed, ‘water stress’ was a major GO category for hypomethylated experiment 1 versus experiment 2 DMRs (8% of the genes, *P*=3.4 × 10^−5^), while ‘tissue development’ was a major GO category for hypermethylated DMRs (9% of the genes, *P=*2.0 × 10^−6^) (Supplementary Fig. S10B, C,; Supplementary Table S5).

Finally, we considered the mapped genes in the 161 DMRs that were common among the three experiments as epigenetic signatures (Fig. 7D). The major identified GO category was ‘response to abiotic stress’ (16% of the genes, *P*=1.70 × 10^−7^; Fig. 8, Supplementary Table S5). We then compared these genes in common DMRs with the DEGs reported for experiment 3 (Lafon-Placette *et al.*, 2018; Fig. 1). Eleven DEGs (Supplementary Table S4) were identified, mostly in hypermethylated regions (Supplementary Fig. S7). Among these genes, *STRUWWELPETER* and *SPINDLY* are already known to be involve in stress and hormone signaling (Autran *et al.*, 2002; Qin *et al.*, 2011; Sarnowska *et al.*, 2013; Wang *et al.*, 2015; Steiner *et al.*, 2016).

To conclude, abiotic-responsive genes (including some hormone-responsive genes) and shoot-development genes showed epigenetic signatures by preferential DNA methylation changes in response to environmental changes. Variations of gene methylation were conserved from active to winter-dormant SAM. The conservation of methylation marks suggests a memory of epigenetic signatures in the SAM that could contribute to stress memory and adaptation of trees. This could suggest a memory of these epigenetic signatures in the SAM contributing to stress memory and adaptation in trees, as proposed in a model shown in Supplementary Fig. S12.

## Discussion

### Global DNA methylation in winter-dormant SAM is a biomarker of genotype performances

Variations in global DNA methylation have been reported in different plant species (Lambé *et al.*, 1997; Alonso *et al.*, 2015; Plomion *et al.*, 2016), within the population (Vaughn *et al.*, 2007; Schmitz *et al.*, 2013) and the offspring of the same parents (Becker *et al.*, 2011; Schmitz *et al.*, 2011; Eichten *et al.*, 2012). Variations in global DNA methylation may also depend on environmental constraints or developmental processes (Causevic *et al.*, 2005; Teyssier *et al.*, 2008; Gourcilleau *et al.*, 2010; Trap-Gentil *et al.*, 2011; Teyssier *et al.*, 2014; Conde *et al.*, 2017). In poplar, variations in global DNA methylation have been reported in response to limited water availability, water or soil salinity, competition, and pathogens, or according to geographic origin, in a limited number of genotypes (Gourcilleau *et al.*, 2010; Raj *et al.*, 2011; Latzel *et al.*, 2013; Garg *et al.*, 2015; Song *et al.*, 2016; Lafon-Placette *et al.*, 2018).

Here, we analyzed global DNA methylation variations in winter-dormant SAMs for two collections of poplar genotypes grown in open fields. The levels of methylation reported here for 39 poplar genotypes were in agreement with previously reported data (Gourcilleau *et al.*, 2010; Raj *et al.*, 2011; Lafon-Placette *et al.*, 2018). The range of variation in our study (~34%) was similar to that reported for *Dianthus broteri* (29–35%; Alonso *et al.*, 2016), a collection of angiosperms (5–40%; Alonso *et al.*, 2015), or for vernalized sugar beet genotypes (20–80%; Trap-Gentil *et al.*, 2011). These results show that DNA sequence variation is not the only determinant of genotypic and probably phenotypic variations; epigenetic variations are also key players (Latzel *et al.*, 2013; Cortijo *et al.*, 2014; Kooke *et al.*, 2015; Kawakatsu *et al.*, 2016; Richards *et al.*, 2017).

In the present study, significant genotype×environment interaction for the global methylation in winter-dormant SAM was identified. In each experiment, correlations of global DNA methylation were established with shoot biomass production. This is in agreement with several studies proposing that variation in DNA methylation has a genetic basis and is a sign of local adaptation (Dubin *et al.*, 2015; Foust *et al.*, 2016).The Arabidopsis 1001 Epigenomes project also provides evidence that methylation is correlated with geography and climate of origin (Kawakatsu *et al.*, 2016). In addition, positive correlations between shoot biomass and DNA methylation have already been reported in a study on active SAM of *P. deltoides* × *P. nigra* genotypes under well-watered conditions (Gourcilleau *et al.*, 2010). A recent study also highlights site-specific growth performance related to methylation patterns in *P. trichocarpa* clones coming from two distinct sites with different phosphorus nutrition; trees growing with adequate phosphorus nutrition had higher dry shoot biomass and global DNA methylation levels (Schönberger *et al.*, 2016). In our study, under favorable growth conditions we identified a positive correlation between fresh shoot biomass and global DNA methylation, while the opposite correlation was detected under unfavorable growth conditions. These results suggest that DNA methylation could be a biomarker of productivity in poplar, at least in the sites analyzed in the present study.

### Winter-dormant SAM keeps the epigenetic memory of environmental variations

DNA methylation plays a role in the ability of plants to acclimate and adapt to changing environmental conditions (Boyko and Kovalchuk, 2008; Bräutigam *et al.*, 2013; Rico *et al.*, 2014; Baulcombe and Dean, 2014; Kawakatsu *et al.*, 2016; Xu *et al.*, 2018; Richards *et al.*, 2017), more specifically, through stress memory (Ding *et al.*, 2014; Kinoshita and Seki, 2014; Meyer, 2015; Yong-Villalobos *et al.*, 2015; Espinas *et al.*, 2016; Fleta-Soriano and Munné-Bosch, 2016; Schönberger *et al.*, 2016; Mauch-Mani *et al.*, 2017). Trees are long-lived organisms that are subjected to repeated environmental constraints; therefore, further studies are needed to better understand environmental epigenetic memory (Allen *et al.*, 2010; Raj *et al.*, 2011; Bräutigam *et al.*, 2013; IPCC, 2014; Schönberger *et al.*, 2016). The best-known example concerns the studies of environmental epigenetic memory in Norway spruce somatic embryos. These studies highlight that environmental epigenetic memory regulates bud phenology and acclimation to the cold (Johnsen *et al.*, 2009; Yakovlev *et al.*, 2010; 2011; 2016; Yakovlev and Fossdal, 2017). Furthermore, an epigenetic memory of temperature during embryogenesis was first observed as important differences in bud phenology in epitypes (Carneros *et al.*, 2017).

Recently, the role of DNA demethylation in poplar SAM was demonstrated for bud burst (Conde *et al.*, 2017). We identified that when short-term variations in water availability occur, poplar active SAM integrates hormonal signals through epigenomic and transcriptomic imprints. These imprints modulate shoot growth and morphogenesis (Lafon-Placette *et al.*, 2018). In this study, we showed that trees grown in favorable or unfavorable open field conditions in two independent experiments still exhibited DMRs in their winter-dormant SAMs 6 months after the summer period. Several reports have shown that the epigenetic machinery in meristematic cells is specific and plays a major role in cell fate (Baubec *et al.*, 2014; Birnbaum and Roudier, 2017). In accordance with these findings, we previously showed that the methylated non-condensed chromatin fraction of active SAM in poplar covered 1.9% of the poplar genome. The non-condensed chromatin fraction is a region where variations in DNA methylation could influence the developmental trajectory. This fraction displayed 74% of the poplar gene models, which were mostly exons (Lafon-Placette *et al.*, 2013). Our results also suggest that the dormant SAM kept the DNA methylation changes that had arisen during the active vegetative period and which correspond to stable epigenetic modifications. Such a phenomenon suggests epigenetic memory. We defined this phenomenon as an epigenetic stress memory that transmits from active SAM in the dividing meristem cells to the dormant cell. The transmission of epigenetic memory has been reported for vernalization in Arabidopsis (Baulcombe and Dean, 2014).

We found that most of the DMRs in the winter-dormant SAM were located in genes (body and promoter). Furthermore, although to a lesser extent, these DMRs were also found in TEs inactivated by hypermethylation; this echoes the findings of Secco *et al.* (2015). Two approaches were then used to characterize the genes covered by DMRs: (i) without any *a priori* considerations, where all the genes in the DMRs were analyzed for GO enrichment, and (ii) with a candidate approach using the available DEGs analysis on poplar active SAMs exposed to different water regimes and being involved in the abiotic stress response (Lafon-Placette *et al.*, 2018). Other transcriptomic data were not used because we have recently shown that transcriptomics in water-stressed SAM are different from those in the leaves or roots (Lafon-Placette *et al.*, 2018). Globally, we found genes that are mostly involved in RNA metabolism, the abiotic stress response, the cell cycle and division, and phytohormone pathways (jasmonic acid, abscisic acid, and salicylic acid). These results explain well our biological context of SAM in changing environments, and are in agreement with a recent study in active SAM showing that DNA methylation preferentially affects phytohormone pathways in response to water availability (Lafon-Placette *et al.*, 2018). Altogether, our data, combined with the study of Lafon-Placette *et al.* (2018), suggest that active SAM integrates environmental variations as DNA methylation in epigenetic footprints. Epigenetic footprints could partially be transmitted mitotically, for several months, up until the winter-dormant stage (Supplementary Fig. S12).

### SAM displays epigenetic signatures in response to environmental variations

Although epigenetics plays a role in stress memory, the ecological significance in terms of acclimation and adaptation requires more evidence. In this context, more field experiments and studies at the population level are necessary to confirm the role of epigenomics in stress memory (Richards *et al.*, 2017). To our knowledge, previous studies on ecophysiological traits and epigenomics related to SAM of poplar clones have been performed in one ecological condition. This study is the first to propose a comparative analysis of ecophysiological traits and epigenomics related to poplar SAM among three ecological conditions (Fig. 1). Our comparative analysis allowed us to identify the loci that were systematically targeted by DNA methylation variations related to site growth performances (called epigenetic signatures) transmitted from active to winter-dormant SAM.

Interestingly, the common DMRs of distinct clones overlapped with certain genes and DEGs (Lafon-Placette *et al.*, 2018). Those genes are involved in abiotic stress response, phytohormone signaling, and shoot meristem development, which can all contribute to short-term acclimation of trees or their resistance to repeated stress (Zhang *et al.*, 2013; Yona *et al.*, 2015; Yamamuro *et al.*, 2016; Richards *et al.*, 2017; Lafon-Placette *et al.*, 2018; Supplementary Fig. S12). In the common DMRs, it was also possible to detect two known poplar water-stress DEGs involved in stress acclimation, growth regulation, cell differentiation and proliferation, and the regulation of hormone signaling. *STRUWWELPETER* encodes a positive regulator of transcription involved in defense signaling crosstalk (salicylic acid and jasmonic acid/ethylene) and *SPINDLY* encodes a regulator of gibberellin and cytokinin signaling that interacts with the chromatin remodeling complex (Autran *et al.*, 2002; Qin *et al.*, 2011; Sarnowska *et al.*, 2013; Wang *et al.*, 2015; Steiner *et al.*, 2016). Our results propose that DNA methylation preferentially affects specific responsive genes and conserves these signatures in the winter-dormant SAM, although several studies have demonstrated the existence of extensive remodeling of DNA methylation in response to stresses. Therefore, a putative selective variation of DNA methylation on stress-responsive genes has been proposed (Colaneri and Jones, 2013; Liang *et al.*, 2014; Garg *et al.*, 2015; Secco *et al.*, 2015; Yong-Villalobos *et al.*, 2015; Wibowo *et al.*, 2016). Our data are also in agreement with two recently published studies on the active SAM of poplar subjected to environmental variations (Conde *et al.*, 2017; Lafon-Placette *et al.*, 2018).

Future studies could use functional analysis to confirm the biological function of these gene categories, which we identified through a GO enrichment approach. In addition, although our comparative analysis was performed on distinct clones and systems of production (see Table 1, Fig. 1, and the Materials and methods for details), a significant number of conserved DMRs were detected. In order to improve comparative analysis and to test the potential ‘priming’ effect (Lämke and Bäurle, 2017) in future investigations, it would be relevant to follow the epigenetic memory and corresponding level of tolerance to abiotic stress. This type of comparative approach has to be managed on a defined set of poplar clones grown under similar management at distinct pedoclimatic sites and analyzed over successive years of growth and in distinct seasons. Another challenge will be to validate the role of the epigenetic signature through a reverse genetic approach with hyper- or hypomethylated RNAi lines (Zhu *et al.*, 2013; Conde *et al.*, 2017; A-L. Le Gac and S. Maury, unpublished results) or through CRISPR/Cas9 technology (Liu *et al.*, 2017, (Fan *et al.*, 2015). It would be relevant to use CRISPR/Cas9 for epigenetic editing for DNA methylation on common DMRs and evaluate the impact on poplar stress memory. Finally, histone modifications, small and long non-coding RNAs, and nucleosome occupancy and chromatin structure should also be investigated to complete our understanding of the epigenetic mechanisms involved in stress memory in trees (Lämke and Bäurle, 2017).

## Supplementary data

Supplementary data are available at *JXB* online.

Table S1. Description of poplar clones used in experiment 2.

Table S2. Gene v3 annotation counts associated with their respective probe category subset.

Table S3. Differentially methylated regions (DMRs) for experiments 1 and 2 and for commonly conserved DMRs.

Table S4. Overlap between genes localized in DMRs and DEGs.

Table S5. Summary of AGRIGO statistical results with the number of genes, *P*-values and FDRs for major Gene Ontology categories of REVIGO treemap analyses.

Fig. S1. Methylome description for an additional unfavorable site in experiment 2, located at Guémené (GMN).

Fig. S2. REVIGO treemap of biological process GO clustering based on the abs log_10_*P*-values of hypo- and hypermethylated probes in DMRs located in the gene body for each condition and experiment.

Fig. S3. REVIGO treemap of biological process GO clustering based on the abs log_10_*P*-value of genes in DMRs in experiment 1 and experiment 2.

Fig. S4. REVIGO treemap of biological process GO clustering based on the abs log_10_*P*-value of genes in DMRs in experiment 1: hypomethylated DMRs and hypermethylated DMRs.

Fig. S5. REVIGO treemap of biological process GO clustering based on the abs log_10_*P*-value of genes in DMRs in experiment 2: hypomethylated DMRs and hypermethylated DMRs.

Fig. S6. REVIGO treemap of biological process GO clustering based on the abs log_10_*P*-value of DEGs in DMRs in experiment 1 or 2.

Fig. S7. DEG counts relative to DMRs: down-regulated DEGs and up-regulated DEGs between favorable and unfavorable growth conditions.

Fig. S8. REVIGO treemap of biological process GO clustering based on the abs log_10_*P*-value of genes in DMRs common to experiments 1 and 3: experiments 1 and 3 (*n*=2029), hypomethylated DMRs, and hypermethylated DMRs between favorable and unfavorable growth conditions.

Fig. S9. REVIGO treemap of biological process GO clustering based on the abs log_10_*P*-value of genes in DMRs common to experiments 2 and 3: experiments 2 and 3 (*n*=2900), hypomethylated DMRs, and hypermethylated DMRs between favorable and unfavorable growth conditions.

Fig. S10. REVIGO treemap of biological process GO clustering based on the abs log_10_*P*-value of genes in DMRs common to experiments 1 and 2: experiments 1 and 2 (*n*=998), hypomethylated DMRs, and hypermethylated DMRs between favorable and unfavorable growth conditions.

Fig. S11. REVIGO treemap of biological process GO clustering based on the abs log_10_*P*-value of genes in DMRs common to experiments 1 and 2: Common conserved variation DMRs and common inversed variation DMRs between favorable and unfavorable growth conditions.

Fig S12. Model for epigenetic environmental memory in the poplar shoot apical meristem.

Method S1. False discovery rate estimation for experiments 1 and 2.

## Supplementary Material

Supplementary Table S1

Supplementary Figures S1-S12, Table S2, Protocol S1

Supplementary Table S3

Supplementary Table S4

Supplementary Table S5
