# Supplementary material for: Winter-dormant shoot apical meristem in poplar trees shows environmental epigenetic memory
Source: J Exp Bot. 2018 Aug 9;69(20):4821–37. doi: 10.1093/jxb/ery271 (PMC6137975; doi:10.1093/jxb/ery271)
Supplement: Supplementary Figures S1-S12, Table S2, Protocol S1 [file ery271_suppl_supplementary_figures_table-s2_protocol-s1.pdf]

Journal of Experimental Botany. **Supporting Information**

Article title: **Winter-dormant shoot apical meristem in poplar trees shows environmental epigenetic memory**

Authors: Anne-Laure Le Gac[1], Clément Lafon-Placette[1,‡], Didier Chauveau[2], Vincent Segura[3], Alain Delaunay[1], Régis Fichot[1], Nicolas Marron[4], Isabelle Le Jan[1], Alain Berthelot[5], Guillaume Bodineau[6], Jean-Charles Bastien[3], Franck Brignolas[1], Stéphane Maury[1,$]

Article acceptance date: Click here to enter a date.

The following Supporting Information is available for this article:

Table S1 Description of poplar clones used in Experiment 2 ("Relationship among the Experiment 2 clones"; Excel file).

Table S2 Gene v3 annotation counts associated with their respective probe category subset. For each gene, the corresponding probes were localized and then classified as "Promoter", "Gene body" or both if overlapping. Numbers in brackets indicate the number of genes common to both Experiments 1 and 2.

	Probes designed for genes in DMRs	Promoter	Gene body	Promoter and Gene body
Experiment 1	87.4%	41 (9)	3466 (906)	95 (13)
Experiment 2	86.1%	71	4869	98

Table S3 Differentially Methylated Regions (DMRs) for Experiments 1, 2 and for commonly conserved DMRs ("Experiment 1_DMRs Experiment 2_DMRs and common_conserved_DMRs"; Excel file).

Table S4 Overlap between genes localized in DMRs and DEGs ("DMRs vs DEGs"; Excel file). Genes localized in 'hypo-methylated' DMRs in Experiment 1 and DEGs; DMRs common to Experiments 1 and 2 and DEGs; and DMRs common to the 3 Experiments and DEGs. DEGs have been recently reported (Lafon-Placette et al., 2018) and are available on GEO GSE46605).

Table S5 Summary of AGRIGO statistical results with the number of genes, p-values and FDRs for major Gene Ontology category of Revigo tree map analyses**.**

Fig. **S1** Methylome description for an additional unfavorable site in Experiment 2, located at Guémené (GMN). (A) Genomic features of DNA methylation changes in the shoot apical meristem represented by Manhattan plot showing?? a significant false discovery rate (FDR) of 5 %. The plots show -log10 P-values on the y-axis and the location of the different 50 kb windows through the genome with a gap at chromosome locations on the x-axis. Blue dots correspond to windows hypo-methylated as compared to the reference mean, red dots to windows hyper-methylated as compared to the reference mean and grey dots to non-significant windows (Methods S1). (B) Characterization of DNA methylation distribution by observing the types of loci affected in the DMRs. DMRs were classified as hypo- or hyper-methylated in a given set of conditions (lower or higher DNA methylation status, than in the other two sets of conditions). BODY: gene body; PROMOTER: 1 kb upstream region; TE: transposable element; BODY+TE: TE inserted into a gene body; PROM+TE: TE inserted into a promoter. INTERGENIC: any other locus. (C) Revigo Tree map of biological process GO clustering based on the abs log10 p-value of shoot apical meristem DNA methylation changes among probes affected. DMRs were classified as hypo- or hyper-methylated in a given set of conditions (lower or higher DNA methylation status, , than in the other two sets of conditions). The representatives of categories are combined into 'superclusters' of loosely related terms, visualized with different colors. Rectangle size is adjusted to reflect the abs log10 p-value of the GO term in the underlying GOA database. 'n' corresponds to homologous *Arabidopsis* gene annotations.

Fig. S2 Revigo Tree map of biological process GO clustering based on the abs log10 p-values (http://revigo.irb.hr/revigo.jsp) of hypo- and hyper-methylated probes in DMRs located in the

gene body for each condition and Experiment. The representatives are combined into 'superclusters' of loosely related terms, visualized with different colors. Rectangle size is adjusted to reflect the abs log10 p-value of the GO term in the underlying GOA database. The number ('n') of homologous *Arabidopsis* gene annotations is indicated in brackets. Experiment 1 conditions are 'ORLWW' and 'ORLWD' and Experiment 2 conditions are 'ECH' Echigey and 'SCV' Saint-Cyr-en-Val.

Fig. S3 Revigo Tree map of biological process GO clustering based on the abs log10 p-value of genes in DMRs in: (A) Experiment 1, (B) Experiment 2. The representatives are combined into 'superclusters' of loosely related terms, visualized with different colors. Rectangle size is adjusted to reflect the abs log10 p-value of the GO term in the underlying GOA database. 'n' corresponds to homologous *Arabidopsis* gene annotations.

Fig. S4 Revigo Tree map of biological process GO clustering based on the abs log10 p-value of genes in DMRs in Experiment 1: (A) Hypomethylated DMRs (B) Hypermethylated DMRs. 1 for hypermethylated, 0 for non-significant and -1 for hypomethylated DNA variation. Each rectangle is a single cluster representative of TAIR10 corresponding poplar V 3.0 annotation. The representatives are combined into 'superclusters' of loosely related terms, visualized with different colors. Size of the rectangles may be adjusted to reflect the abs log10 p-value of the GO term in the underlying GOA database. 'n' corresponds to homologous *Arabidopsis* gene annotations.

Fig. S5 Revigo Tree map of biological process GO clustering based on the abs log10 p-value of genes in DMRs in Experiment 2: (A) Hypo-methylated DMRs (B) Hyper-methylated DMRs. 1 for hyper-methylated, 0 for non-significant and -1 for hypo-methylated DNA variations. Each rectangle is a single cluster representative of corresponding TAIR10 poplar V 3.0 annotation. The representatives are combined into 'superclusters' of loosely related terms, visualized with different colors. Rectangle size is adjusted to reflect the abs log10 p-value of the GO term in the underlying GOA database. 'n' corresponds to homologous *Arabidopsis* gene annotations.

Fig. S6 Revigo Tree map of biological process GO clustering based on the abs log10 p-value of DEGs in DMRs in Experiment 1 or 2. DEGs were recently described in Lafon-Placette (2017) through transcriptomic array analysis. Each rectangle is a single cluster representative of corresponding TAIR10 poplar V 3.0 annotation. The representatives are combined into 'superclusters' of loosely related terms, visualized with different colors. Rectangle size is adjusted to reflect the abs log10 p-value of the GO term in the underlying GOA database. 'n' corresponds to homologous *Arabidopsis* gene annotations.

Fig. S7 DEG counts relative to DMRs: (A) Down-regulated DEGs, (B) Up-regulated DEGs between favorable and unfavorable growth conditions. Hypo- and hyper-methylated DMRs between favorable and unfavorable growth conditions are indicated for Experiments 1 and 2 separately, and 1-2-3 combined (mentioned as 'all'). DEGs were reported recently (Lafon-Placette et al., 2018) in SAM under favorable growth conditions (well-watered) and unfavorable growth conditions (water deficit or water deficit followed by re-watering).

Fig. S8 Revigo Tree map of biological process GO clustering based on the abs log10 p-value of genes in DMRs 'common' to Experiments 1 and 3: **(A) Experiments 1 and 3 (n= 2,029),** (B) Hypo-methylated DMRs (C) Hyper-methylated DMRs between favorable and unfavorable growth conditions. Scores for DNA variation are indicated as 1 for hyper-methylated, 0 for non-significant and -1 for hypo-methylated. 'Common' DMRs means that the same locus was detected as a DMR between favorable and unfavorable growth conditions in Experiment 1 and Experiment 3. Each rectangle is a single cluster representative of corresponding TAIR10 poplar V 3.0 annotation. The representatives are combined into 'superclusters' of loosely related terms, visualized with different colors. Rectangle size is adjusted to reflect the abs log10 p-value of the GO term in the underlying GOA database. 'n' corresponds to homologous *Arabidopsis* gene annotations.

Fig. S9 Revigo Tree map of biological process GO clustering based on the abs log10 p-value of genes in DMRs 'common' to Experiments 2 and 3: **(A) Experiments 2 and 3 (n= 2,900),** (B) Hypo-methylated DMRs (C) Hyper-methylated DMRs between favorable and unfavorable growth conditions. Scores for DNA variation are indicated as 1 for hyper-methylated, 0 for non-

significant and -1 for hypo-methylated. ‘Common’ DMRs means that the same locus was detected as a DMR between favorable and unfavorable growth conditions in Experiment 2 and Experiment 3. Each rectangle is a single cluster representative of the corresponding TAIR10 poplar V 3.0 annotations. The representatives are joined into ‘superclusters’ of loosely related terms, visualized with different colors. Rectangle size is adjusted to reflect the abs log10 p-value of the GO term in the underlying GOA database. ‘n’ corresponds to homologous *Arabidopsis* gene annotations.

Fig. S10 Revigo Tree map of biological process GO clustering based on the abs log10 p-value of genes in DMRs ‘common’ to Experiments 1 and 2: **(A) Experiments 1 and 2 (n= 998),** (B) Hypo-methylated DMRs (C) Hyper-methylated DMRs between favorable and unfavorable growth conditions. Scores for DNA variation are indicated as 1 for hyper-methylated, 0 for non-significant and -1 for hypo-methylated. ‘Common’ DMRs means that the same locus was detected as DMR between favorable and unfavorable growth conditions in Experiment 1 and Experiment 2. Each rectangle is a single cluster representative of TAIR10 corresponding to poplar V 3.0 annotation. The representatives are joined into ‘superclusters’ of loosely related terms, visualized with different colors. Rectangle size is adjusted to reflect the abs log10 p-value of the GO term in the underlying GOA database. ‘n’ corresponds to homologous *Arabidopsis* gene annotations.

Fig. S11 Revigo Tree map of biological process GO clustering based on the abs log10 p-value of genes in DMRs ‘common’ to Experiments 1 and 2: (A) ‘Common conserved’ variation DMRs (B) ‘Common inversed’ variation DMRs between favorable and unfavorable growth conditions. Scores for DNA variation are indicated as 1 for hyper-methylated, 0 for non-significant and -1 for hypo-methylated. ‘Common’ DMRs means that the same locus was detected as DMR between favorable and unfavorable growth conditions in Experiment 1 and Experiment 2. ‘Conserved’ versus ‘inversed’ mean that the direction of variation (hypo- or hyper-methylation) of the ‘common’ DMRs between favorable and unfavorable conditions is identical (‘conserved’) or different (‘inversed’) between Experiments 1 and 2. Each rectangle is a single cluster representative of TAIR10 corresponding to poplar V 3.0 annotation. The representatives are joined into ‘superclusters’ of loosely related terms, visualized with different colors. Rectangle

size is adjusted to reflect the abs log10 p-value of the GO term in the underlying GOA database. ‘n’ corresponds to homologous *Arabidopsis* gene annotations.

Figure S12: Model for epigenetic environmental memory in the poplar Shoot Apical Meristem (SAM). Differentially Methylated Regions (DMRs) described in the active SAM between favorable and unfavorable growing conditions during the vegetative period (Lafon-Placette *et al.*, 2018) could be detected in winter dormant SAM (present study) suggesting mitotic transmission. DRMs were shown to preferentially affect genes responding to abiotic stress. Potentially, some of the DMRs playing a role in immediate phenotypic plasticity could be maintained during the next vegetative period and may contribute to acclimation and stress memory (priming); they may also be transmitted to the next generation resulting in trans-generational stress memory and adaptive potential.

Methods S1 FDR estimation for Experiments 1 and 2. This document describes the statistical study conducted to estimate the False Discovery Rate (FDR) of the multiple tests performed for Experiments 1 and 2, as recently reported in Lafon-Placette et al. (2018): (i) estimation of reference means calculated with mixture models on a subsample; (ii) multiple tests with FDR control per window and scaffold; (iii) mixture-based clustering of window means, to compare with results from the FDR procedure; (iv) computation of the Differentially Methylated Regions (DMR). A data frame summarizing all the results per window for each scaffold is produced and some additional summary plots are displayed.

Fig. S1

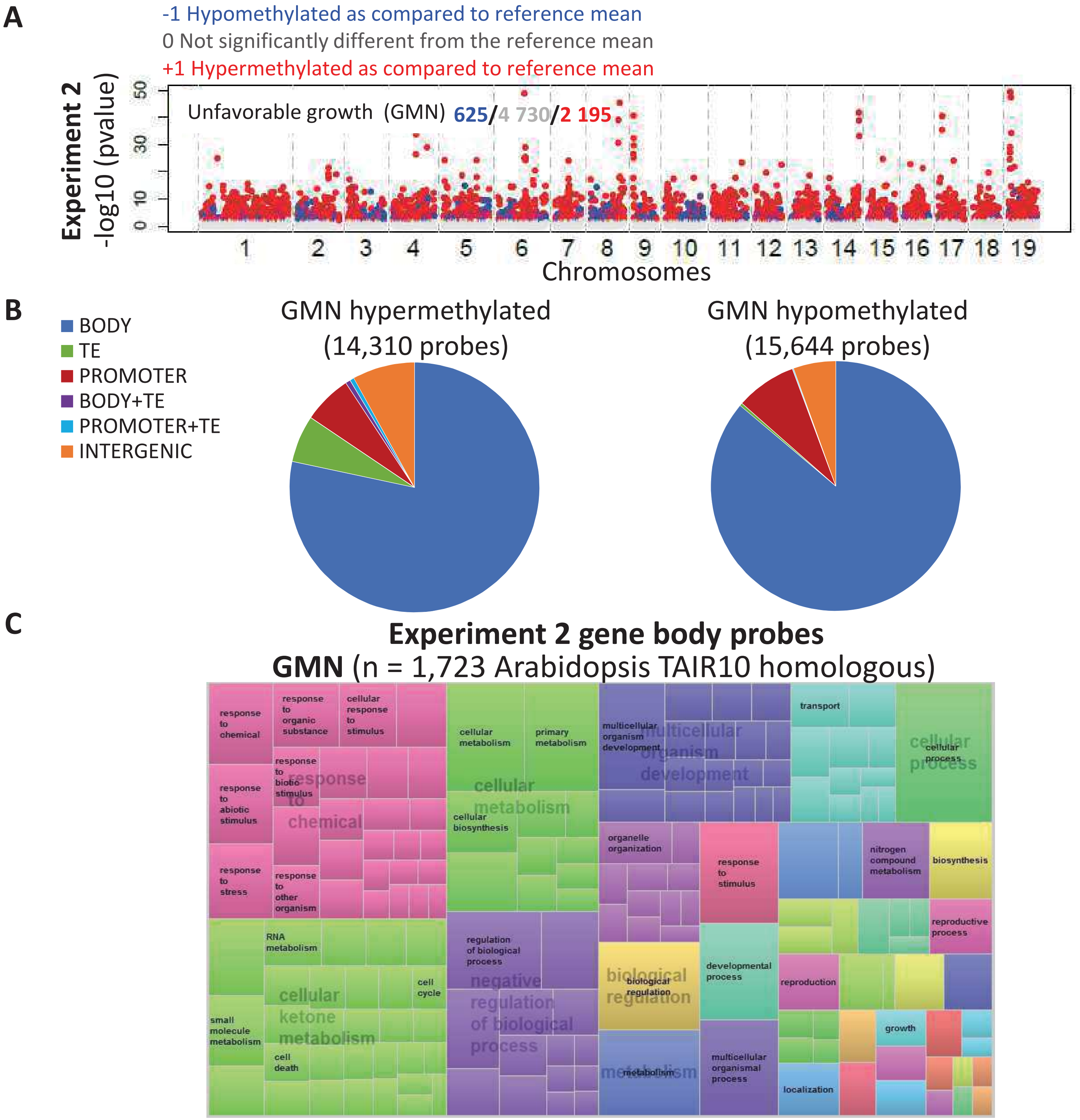
A
-1 Hypomethylated as compared to reference mean
0 Not significantly different from the reference mean
+1 Hypermethylated as compared to reference mean
Experiment 2
-log10 (pvalue)
Unfavorable growth (GMN) 625/4 730/2 195
Chromosomes
1 2 3 4 5 6 7 8 9 10 11 12 13 14 15 16 17 18 19
B
BODY
TE
PROMOTER
BODY+TE
PROMOTER+TE
INTERGENIC
GMN hypermethylated (14,310 probes)
GMN hypomethylated (15,644 probes)
C
Experiment 2 gene body probes
GMN (n = 1,723 Arabidopsis TAIR10 homologous)
response to chemical
response to organic substance
cellular response to stimulus
response to biotic stimulus
response to abiotic stimulus
response to stress
response to other organism
cellular metabolism
primary metabolism
cellular biosynthesis
multicellular organism development
transport
cellular process
organelle organization
response to stimulus
nitrogen compound metabolism
biosynthesis
reproductive process
RNA metabolism
cell cycle
cellular ketone metabolism
small molecule metabolism
cell death
regulation of biological process
negative regulation of biological process
biological regulation
developmental process
reproduction
growth
metabolism
multicellular organismal process
localization

Fig. S2

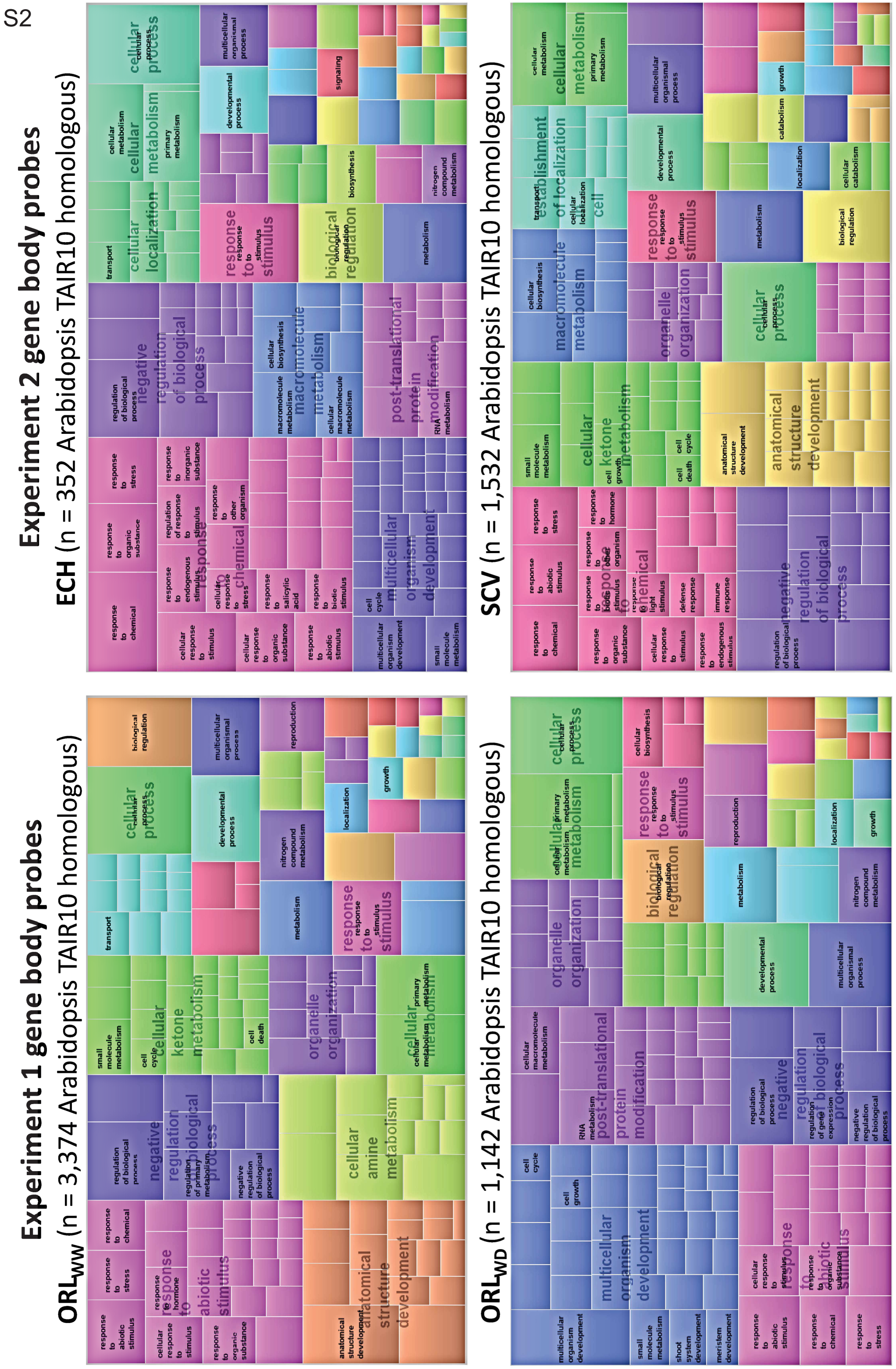

Experiment 2 gene body probes
ECH (n = 352 Arabidopsis TAIR10 homologous)
SCV (n = 1,532 Arabidopsis TAIR10 homologous)
Experiment 1 gene body probes
ORLWW (n = 3,374 Arabidopsis TAIR10 homologous)
ORLWD (n = 1,142 Arabidopsis TAIR10 homologous)

Fig. S3

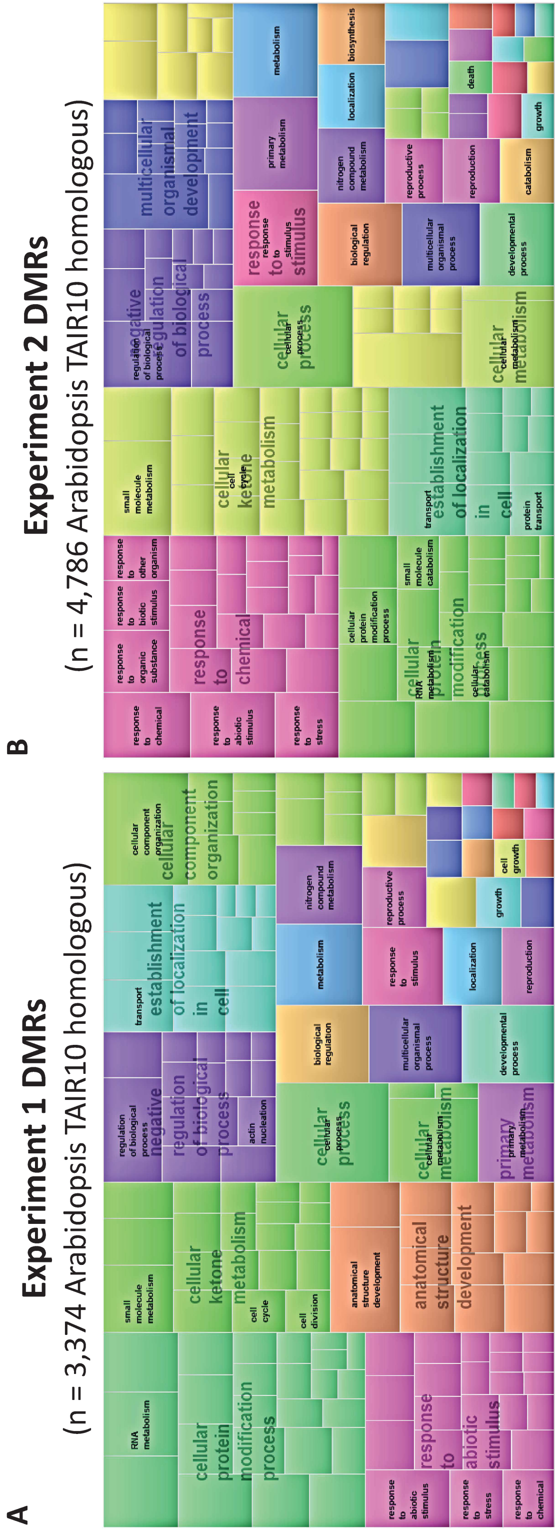
A
Experiment 1 DMRs
(n = 3,374 Arabidopsis TAIR10 homologous)
RNA metabolism
cellular protein modification process
small molecule metabolism
cellular ketone metabolism
cell cycle
cell division
anatomical structure development
response to abiotic stimulus
response to stress
response to chemical
regulation of biological process
negative regulation of biological process
actin nucleation
cellular process
cellular metabolism
primary metabolism
transport
establishment of localization in cell
biological regulation
multicellular organismal process
developmental process
metabolism
response to stimulus
localization
reproduction
nitrogen compound metabolism
reproductive process
growth
cell growth
cellular component organization
B
Experiment 2 DMRs
(n = 4,786 Arabidopsis TAIR10 homologous)
response to chemical
response to organic substance
response to biotic stimulus
response to other organism
response to abiotic stimulus
response to stress
cellular protein modification process
RNA metabolism
small molecule catabolism
cellular catabolism
small molecule metabolism
cellular ketone metabolism
transport
establishment of localization in cell
protein transport
regulation of biological process
negative regulation of biological process
cellular process
cellular metabolism
multicellular organismal development
response to stimulus
primary metabolism
metabolism
biological regulation
nitrogen compound metabolism
localization
biosynthesis
multicellular organismal process
developmental process
reproductive process
reproduction
death
catabolism
growth

Fig. S4

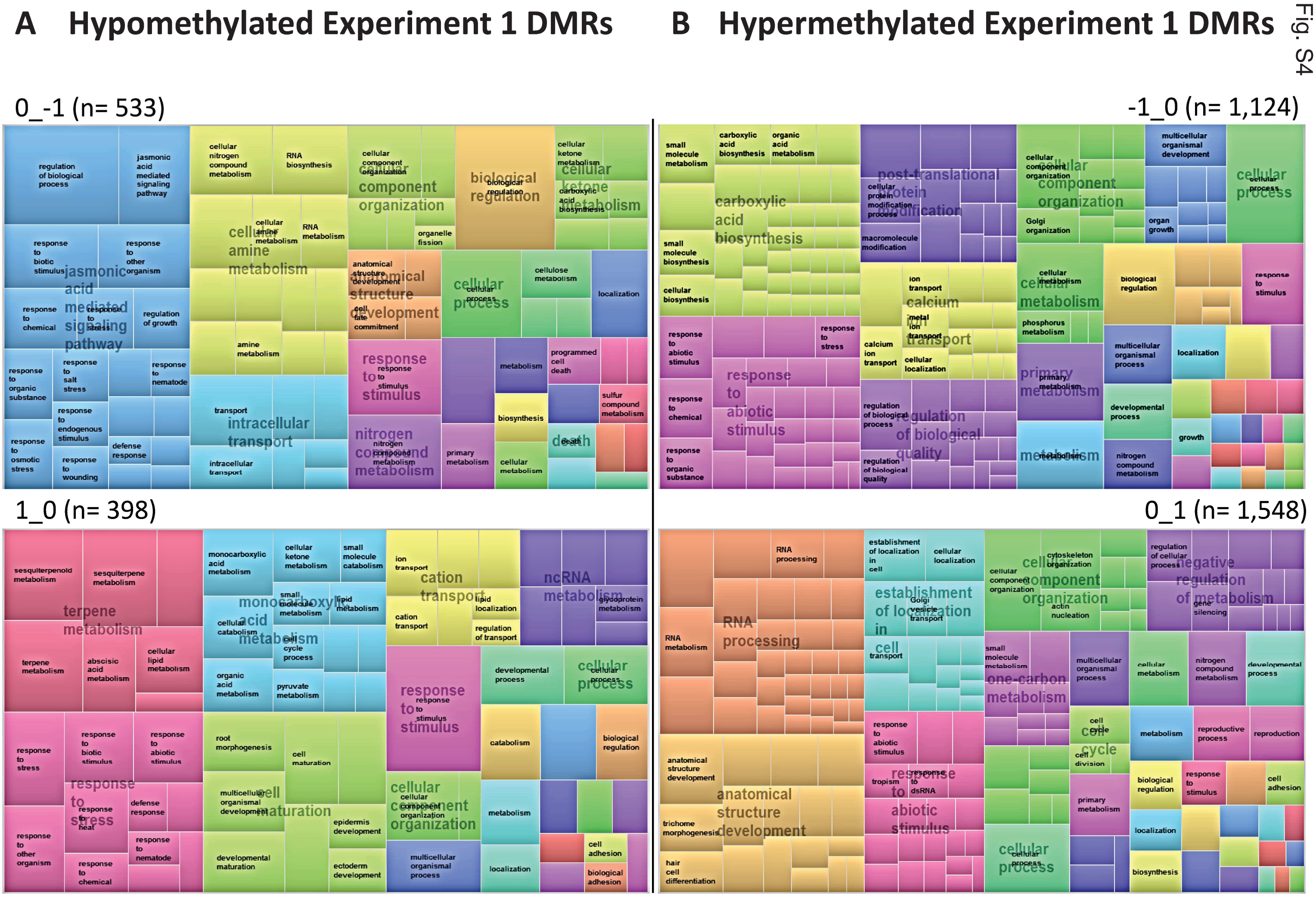
A Hypomethylated Experiment 1 DMRs
B Hypermethylated Experiment 1 DMRs
0_-1 (n= 533)
-1_0 (n= 1,124)
1_0 (n= 398)
0_1 (n= 1,548)
jasmonic acid mediated signaling pathway
cellular amine metabolism
cellular component organization
biological regulation
cellular ketone metabolism
anatomical structure development
cellular process
response to stimulus
nitrogen compound metabolism
intracellular transport
death
terpene metabolism
monocarboxylic acid metabolism
cation transport
ncRNA metabolism
response to stress
cell maturation
post-translational protein modification
carboxylic acid biosynthesis
calcium ion transport
cellular metabolism
primary metabolism
metabolism
regulation of biological quality
response to abiotic stimulus
RNA processing
establishment of localization in cell
cell transport
one-carbon metabolism
negative regulation of metabolism
cell cycle
anatomical structure development

Fig. S5

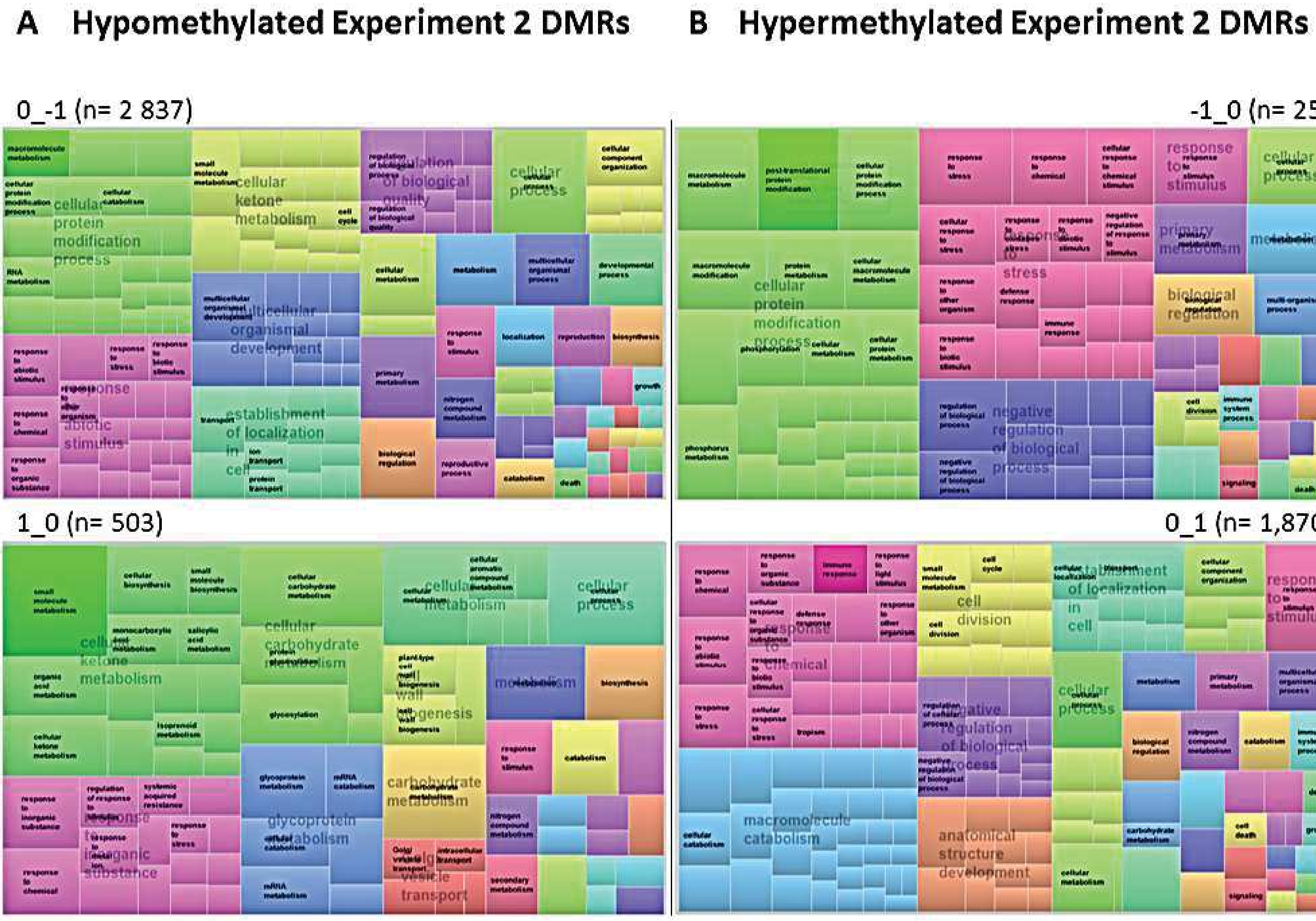
A Hypomethylated Experiment 2 DMRs
B Hypermethylated Experiment 2 DMRs
0_-1 (n= 2 837)
-1_0 (n= 252)
1_0 (n= 503)
0_1 (n= 1,870)
cellular protein modification process
cellular ketone metabolism
regulation of biological quality
cellular process
multicellular organismal development
response to abiotic stimulus
establishment of localization in cell
cellular metabolism
metabolism
multicellular organismal process
developmental process
response to stimulus
localization
reproduction
biosynthesis
primary metabolism
nitrogen compound metabolism
biological regulation
reproductive process
catabolism
death
growth
cellular carbohydrate metabolism
cellular metabolism
wall biogenesis
carbohydrate metabolism
glycoprotein catabolism
Golgi vesicle transport
response to inorganic substance
secondary metabolism
response to chemical
response to stress
macromolecule metabolism
post-translational protein modification
phosphorus metabolism
negative regulation of biological process
response to stimulus
primary metabolism
biological regulation
multi-organism process
immune system process
signaling
cell division
macromolecule catabolism
anatomical structure development
establishment of localization in cell
immune response
tropism
cellular catabolism

Fig. S6

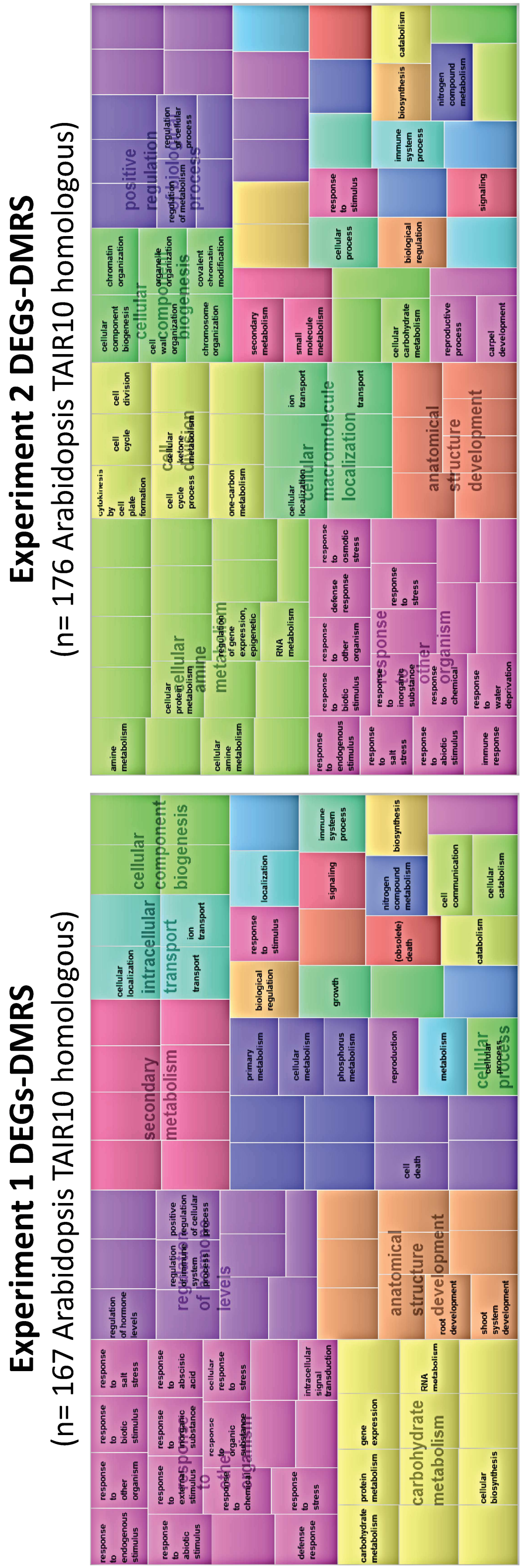
Experiment 2 DEGs-DMRS
(n= 176 Arabidopsis TAIR10 homologous)
positive regulation of biological process
cellular component biogenesis
cell cycle
cellular macromolecule localization
anatomical structure development
cellular amine metabolism
response to other organism
Experiment 1 DEGs-DMRS
(n= 167 Arabidopsis TAIR10 homologous)
cellular component biogenesis
intracellular transport
secondary metabolism
cellular process
anatomical structure development
carbohydrate metabolism

Fig. S7

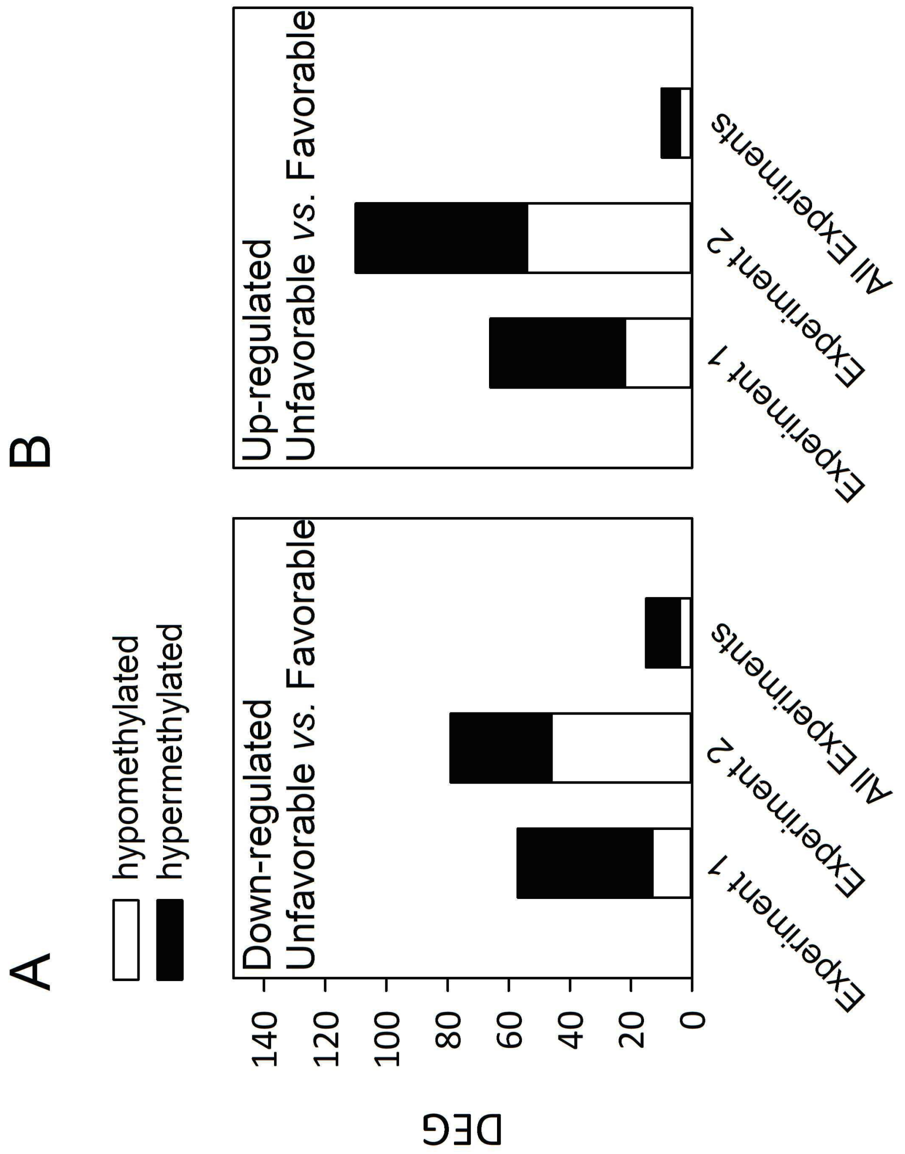
A
B
hypomethylated
hypermethylated
Down-regulated
Unfavorable vs. Favorable
Up-regulated
Unfavorable vs. Favorable
DEG
140
120
100
80
60
40
20
0
Experiment 1
Experiment 2
All Experiments

Fig. S8

A

Experiment 1 and Experiment 3 commons DMRs

(n= 2 029)

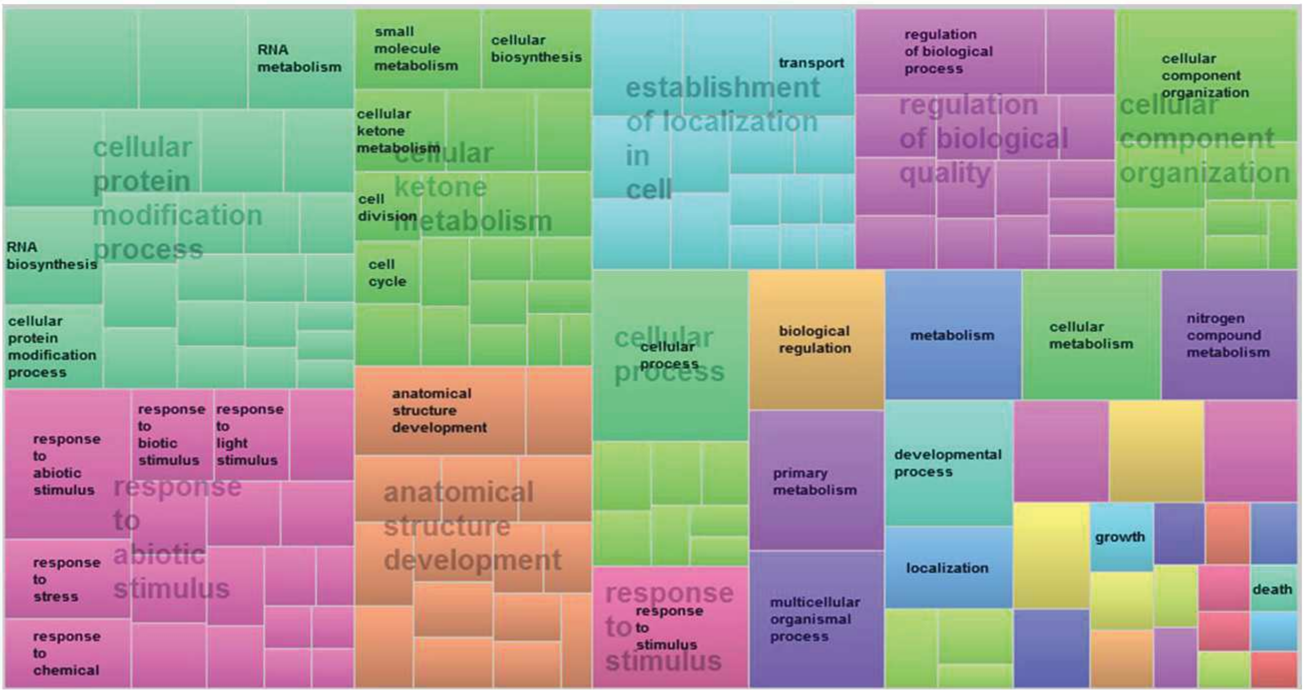

Fig. S8

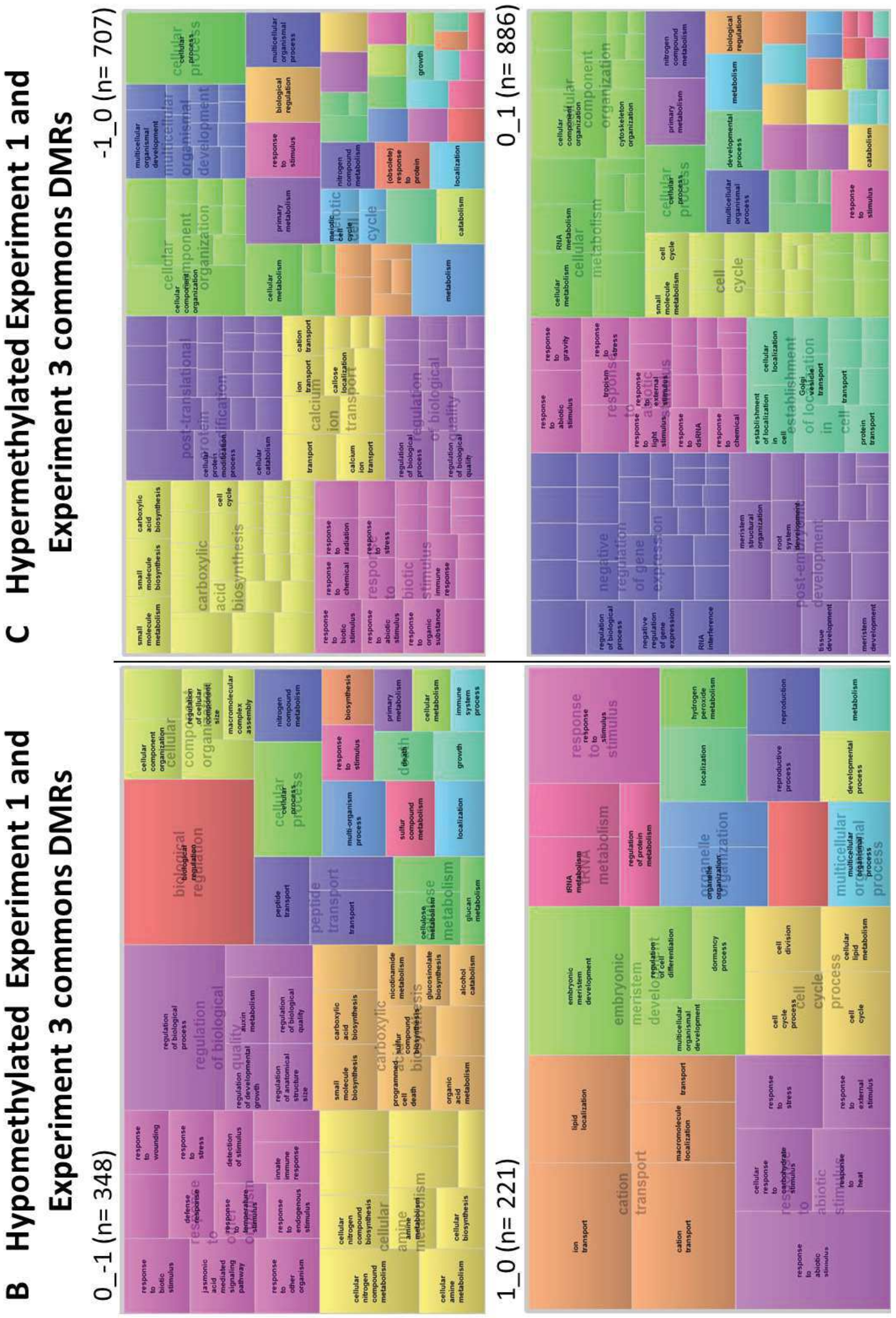
B Hypomethylated Experiment 1 and Experiment 3 commons DMRs
0_-1 (n= 348)
1_0 (n= 221)
C Hypermethylated Experiment 1 and Experiment 3 commons DMRs
-1_0 (n= 707)
0_1 (n= 886)

Fig. S9

A

(n= 2,900)

Experiment 2 and
Experiment 3 commons DMRs

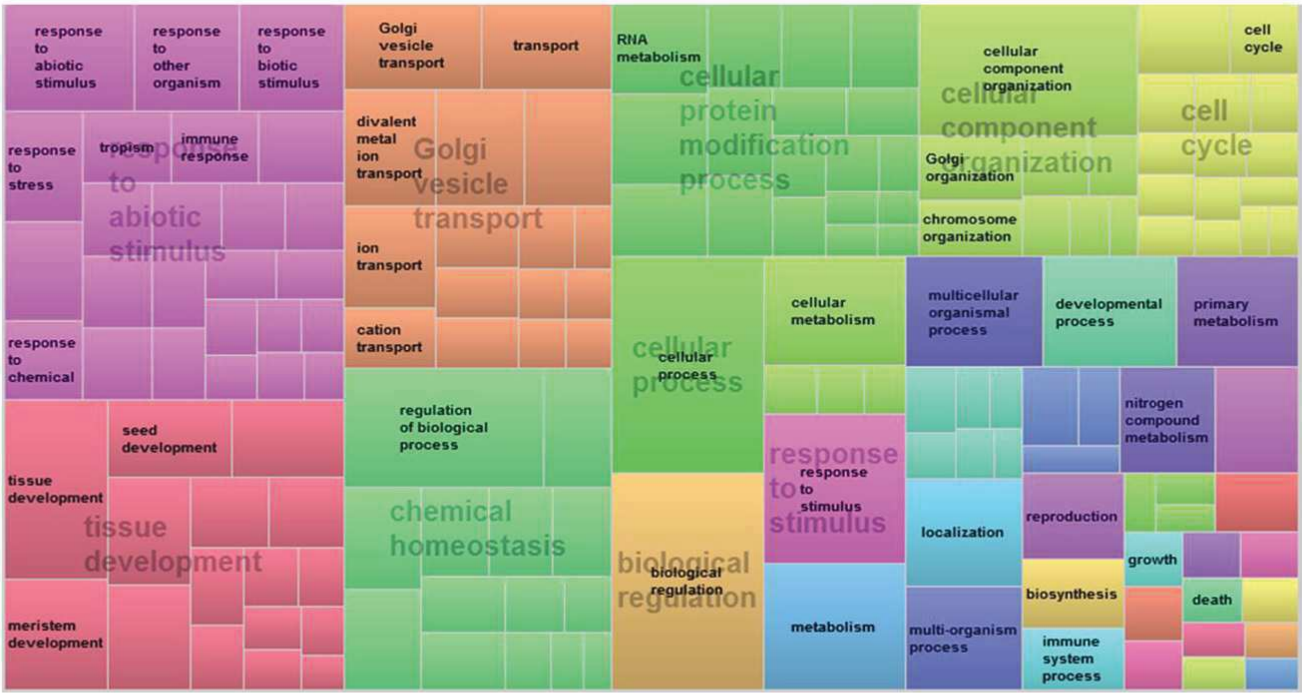

Fig. S9

B Hypomethylated Experiment 2 and Experiment 3 commons DMRs

C Hypermethylated Experiment 2 and Experiment 3 commons DMRs

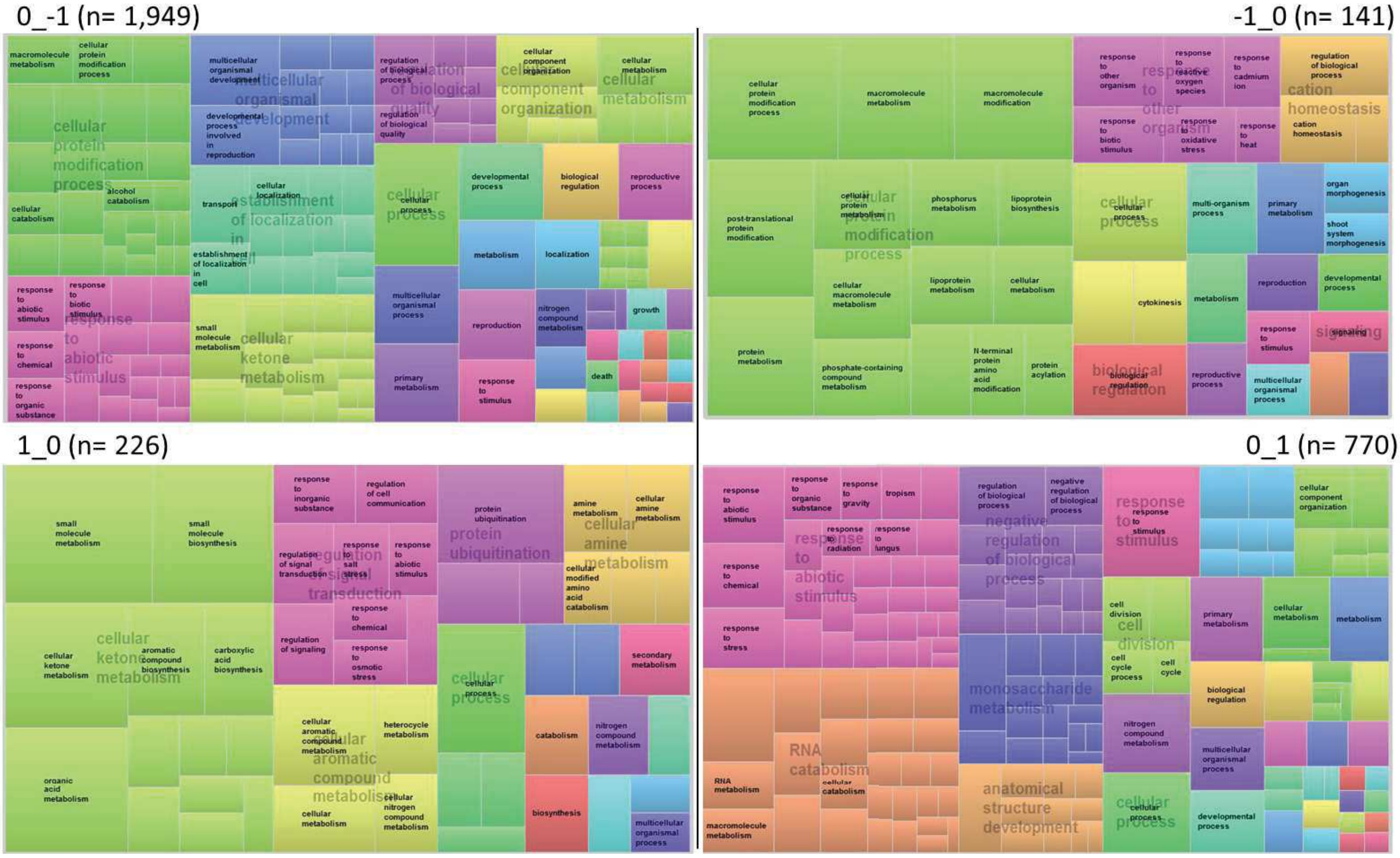

Fig. S10

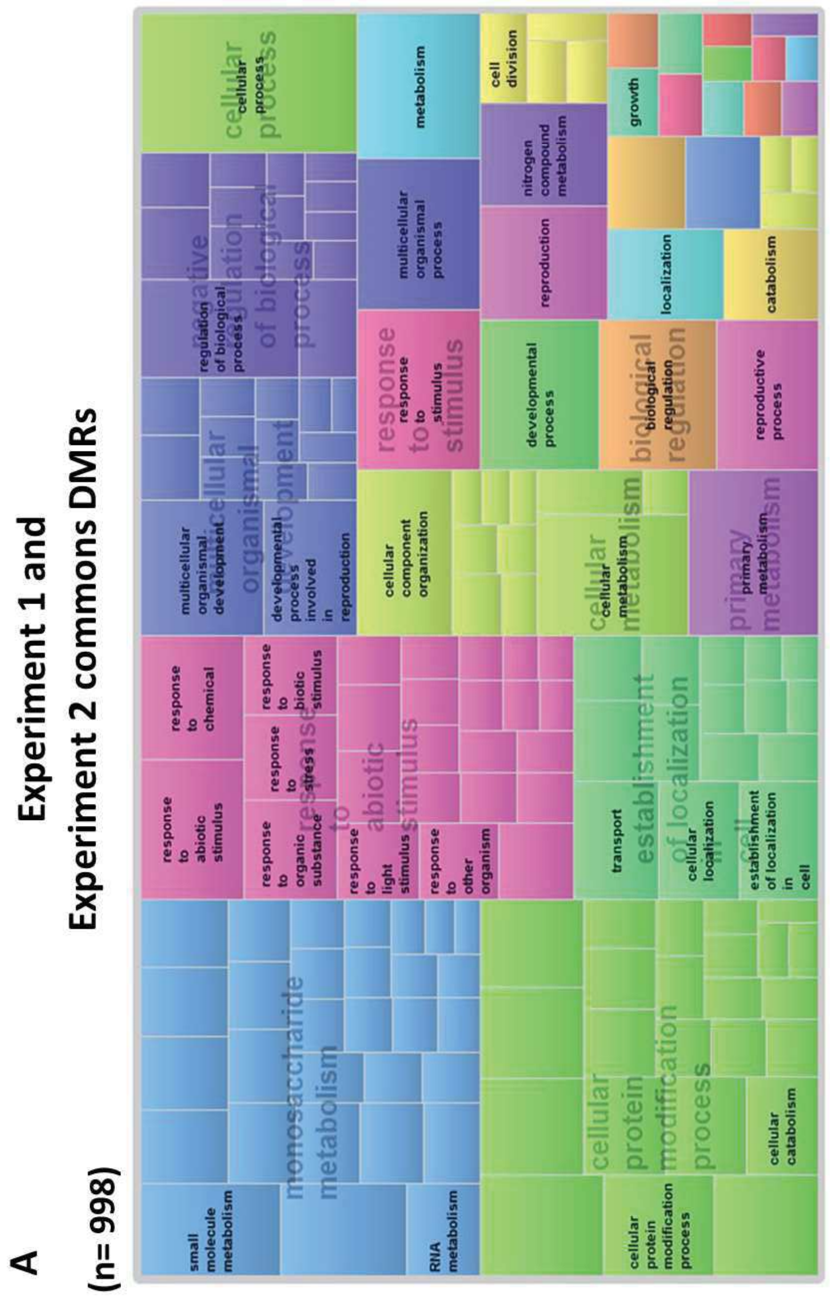
A
(n= 998)
Experiment 1 and
Experiment 2 commons DMRs
small molecule metabolism
monosaccharide metabolism
RNA metabolism
response to abiotic stimulus
response to chemical
response to organic substance
response to stress
response to biotic stimulus
response to light stimulus
response to other organism
response to abiotic stimulus
multicellular organismal development
developmental process involved in reproduction
regulation of biological process
negative regulation of biological process
cellular process
metabolism
multicellular organismal process
response to stimulus
cellular component organization
cellular metabolism
cell division
nitrogen compound metabolism
reproduction
developmental process
biological regulation
growth
localization
catabolism
reproductive process
primary metabolism
transport
establishment of localization
cellular localization
establishment of localization in cell
cellular protein modification process
cellular catabolism

Fig. S10

B Hypomethylated Experiment 1 and Experiment 2 commons DMRs

C Hypermethylated Experiment 1 and Experiment 2 commons DMRs

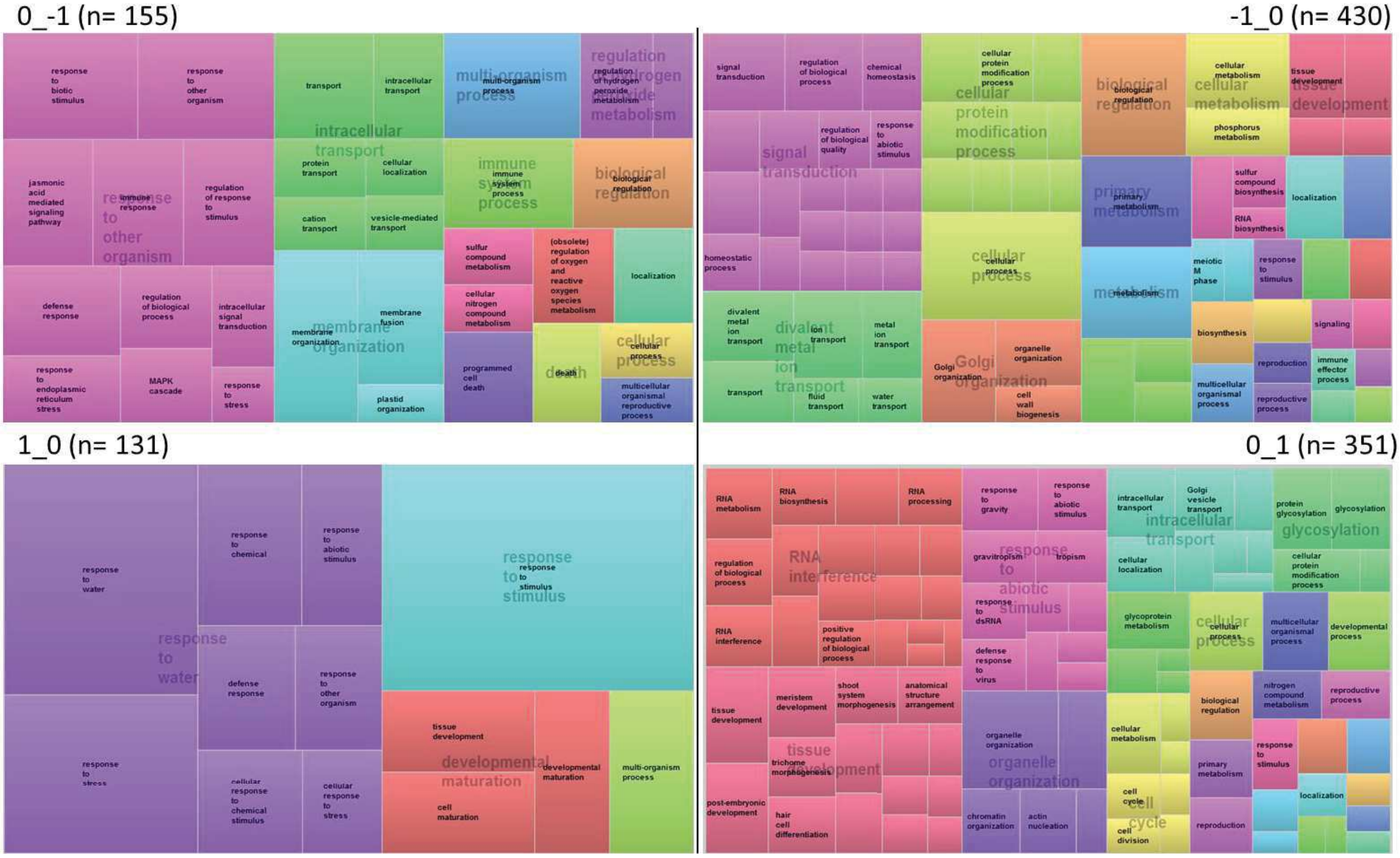

Fig. S11

Common conserved variation DMRs
Experiment 1 and Experiment 2
(n= 326 Arabidopsis TAIR10 homologous)

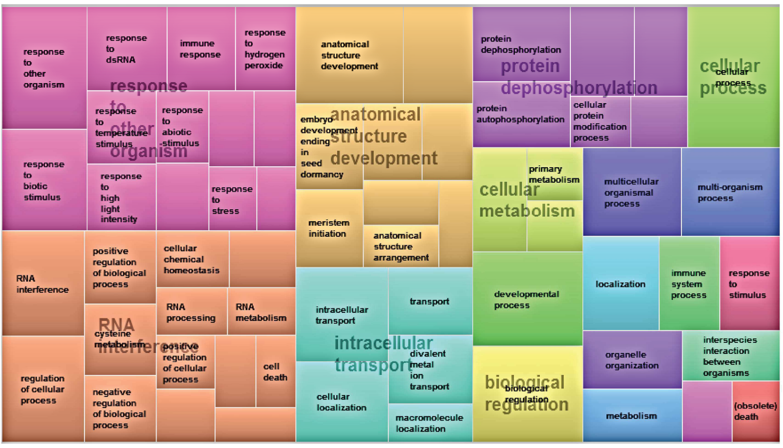

Common inversed variation DMRs
Experiment 1 and Experiment 2
(n= 726 Arabidopsis TAIR10 homologous)

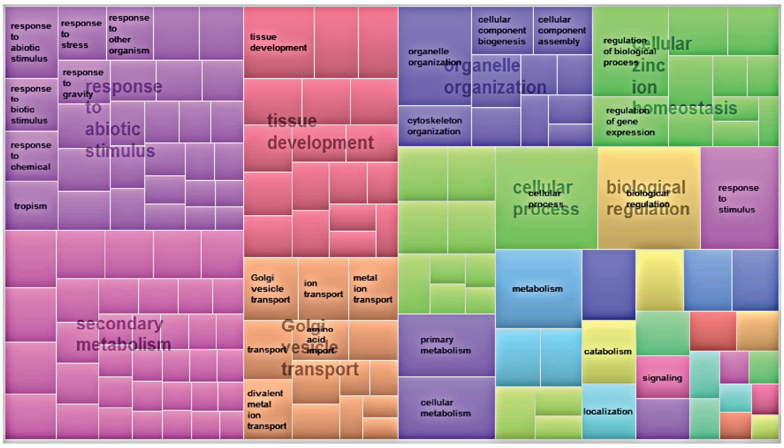

Fig. S12

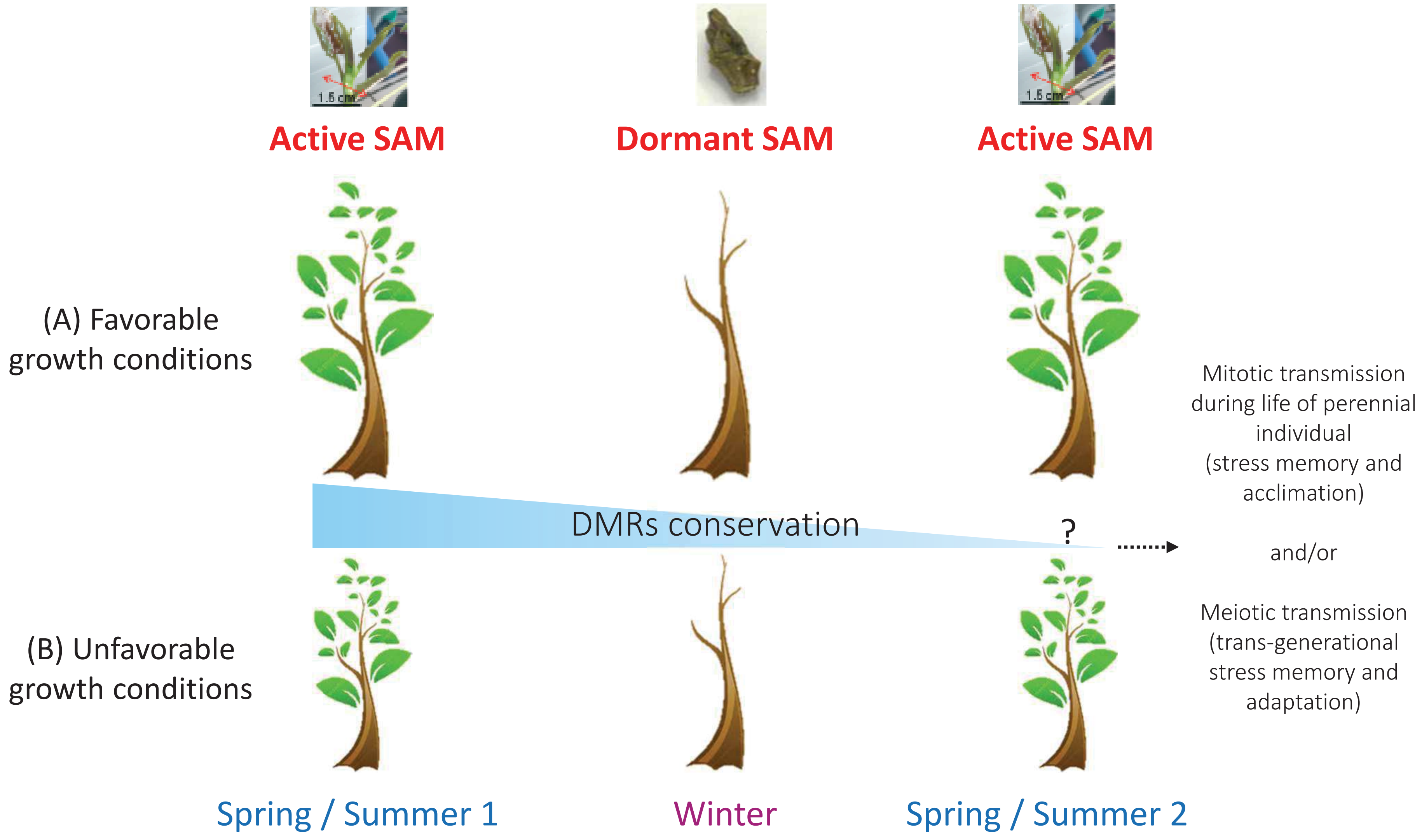

1.5 cm
1.5 cm
Active SAM
Dormant SAM
Active SAM
(A) Favorable growth conditions
(B) Unfavorable growth conditions
DMRs conservation
?
Mitotic transmission during life of perennial individual (stress memory and acclimation)
and/or
Meiotic transmission (trans-generational stress memory and adaptation)
Spring / Summer 1
Winter
Spring / Summer 2

Mixture models and False Discovery Rate (FDR) estimation

Abstract

This document describes in details the statistical study conducted for estimating the False Discovery Rate (FDR) of the multiple tests performed for both Experiments 1 and 2: (i) estimation of reference means based on mixture models on a subsample; (ii) multiple tests with FDR control per windows and scaffolds; (iii) mixture-based clustering of window means, for comparisons with results from the FDR procedure; (iv) computation of the Differentially Methylated Regions (DMR). Some additional summary plots are displayed.

1 Experiment 1: ORL$_{WW}$ and ORL$_{WD}$

1.1 Determination of reference means from Gaussian mixtures

The purpose of this preliminary step is to obtain "reference means" for each of the conditions (called treatments), and for all or each scaffold. The two responses for this experiment are ORL_{WW} and ORL_{WD} (see the main document). Denote μ_0^t this reference mean for treatment t, against which the mean response for each window w, that we denote μ_w, will be compared using a statistical test for a null hypothesis such as $H_0 : \mu_w = \mu_0^t$ in the parametric case, or distribution equality in nonparametric cases (see below). To insure independence between the mean responses per window and the reference mean (since both need to be estimated from the data), we first randomly select 20% of the data as a "reference sample", $n = 61,439$ responses. The remaining dataset ($n = 245,756$) is used for building the multiple test procedure.

We consider the responses for *each* treatment separately, i.e. univariate models only. The plots of empirical distributions (histograms in Fig.1) show that 2-component mixture models are suitable (see McLachlan and Peel, 2000, for references on mixture models). We further assume here that the responses for each treatment form an iid sample from a $m = 2$ component univariate Gaussian mixture. Informally, the distribution associated to each treatment t is given by $\lambda_1^t \mathcal{N}(\mu_1^t, v_1^t) + (1 - \lambda_1^t)\mathcal{N}(\mu_2^t, v_2^t)$, where the statistical model parameters are component 1 weight λ_1^t, component means (μ_1^t, μ_2^t) and variances (v_1^t, v_2^t), hence 5 scalar parameters per treatment. Other mixture models, e.g., nonparametric as in Bordes et al. (2007) or Chauveau et al. (2015) could be tried as well.

For each treatment, the parameters of the model are then estimated using a standard EM algorithm. We use here the normalmixEM function of the Benaglia et al. (2009) mixtools package for the R statistical software (R Core Team, 2016). The initialization of the EM algorithm is data-driven, based on an initial k-means clustering of the data to which we provide initial centroïd corresponding approximately to the modes visible in the histograms. Alternative initialization methods have also been checked to insure that the estimates correspond to the maximum of the loglikelihood of the model for these data.

Fig.1 displays the mixture model fits together with the empirical distributions. The estimated mean of the leftmost, smallest component mean labeled “1” here and associated to the larger estimated weight $\hat{\lambda}_1^t$, is assumed to be the reference for each treatment “t”. Results for the reference means $\hat{\mu}_0^t$ are indicated on Fig.1 and recalled below together with the corresponding proportions (weights) of the leftmost component:

```
                  ORL_WD  ORL_WW
component 1 mean  -0.426  -0.435
      weight (%)  69.3    72.5
```

These estimates are computed on the data from all the 19 scaffolds. We have also fit mixture models for each scaffold separately. Fig.2 shows that, for the 2 treatments, the reference means of the leftmost component per scaffold are comparable to the global reference.

1.2 Multiple tests on consecutive windows

We assume that the μ_0^t's estimated in Section 1.1 from the 20% reference sample provide reference mean parameters that are now considered non-random. The next step consists in building windows of consecutive observations (spots) for some given “nominal” size, and to test equality of means or distributions between each window and the reference. For a nominal size set to 50 kb (see the main document), we obtain the configuration of the windows per scaffold summarized in Fig. 3. More precisely, Fig. 4 illustrates typical responses for one treatment, ORL_{WD}, Scaffold 1, and the first ten windows $w = 1, \ldots, 10$. This shows that window responses may vary in dispersion (as e.g., between scaffolds 3 and 5) or/and in localization (as e.g., between scaffolds 4 and 10), and that the null hypothesis of mean equality may be accepted even though the spots in a window are not close to the reference mean, because of averaging.

1.2.1 Statistical tests per window

In view of some boxplots in Fig. 4, distribution within windows often show some skewness (e.g., window 2), and heavy tails. Hence it seems not reasonnable to assume Gaussian distributions of responses within each window, so that Student t-tests cannot be used. When the number of observations per window is large enough ($n_w \geq 30$ would be typically acceptable) tests using the asymptotic normality (also known as z-tests) may be used. But in most windows, depending on the nominal size, there are fewer obervations, so that nonparametric framework must be used, at least in these cases (for the nominal size 50 kb, about 56% of the windows have fewer than 30 observations). A standard test for this situation is the Wilcoxon signed rank test of a null hypothesis that the distribution of the sample from each window is symmetric about μ_0^t. This test can be viewed as a nonparametric version of the t- or z-tests for localization. However, note that this test may reject (i.e. declare significant) windows appropriately centered toward the reference mean (i.e. for which $\hat{\mu}_w \approx \mu_0^t$), in case of lack of symmetry (skewness). In the sequel, we only apply Wilcoxon signed rank tests on all windows for simplicity (parametric and nonparametric tests per windows could also be mixed, depending on the number of observations per window).

1.2.2 FDR control

The purpose of the study is to declare as significant the windows (i.e. the spots within the windows) for which the null hypotheses have been rejected, according to the p-values of the

tests. As stated before, rejection means significant shift (or non symmetry) from the reference distribution located on μ_0^t for each treatment t. For each scaffold, this experiment results in multiple hypothesis testing problem with hundreds of test responses to consider simultaneously (see Fig. 3, top, for the number of windows per scaffold; for instance the largest configuration with windows of size 50 is 965 windows for scaffold 1). Note also that decreasing the nominal window size (e.g., from 50 kb to 20 kb) results in a larger number of windows per scaffold, but with less observations per window, resulting in less powerful tests.

In this multiple inference setup, the unguarded use of single-inference procedures may results in an increased false positive rate among the simultaneous tests. The first criterion was the FamilyWise Error Rate (FWER), that is the probability of observing at least one false rejection among the n tests. The historical approach since the early 1950s, called the Bonferroni approximation procedure (see e.g. Benjamini and Hochberg (1995) and references therein) consists in applying each test at a level α/n, resulting in a FWER lesser than α. However, Bonferroni-type procedures appear to be far too conservative when n gets large because α/n gets too small, leading to too few rejections. Since then, the point of view on the problem has changed, focusing in the number (or ratio) of erroneous rejections instead of controlling the FWER. In this vein, the most common concept of error control in such multiple testing inference is the expected proportion of falsely rejected hypotheses among the simultaneous tests, or False Discovery Rate (FDR, Benjamini and Hochberg, 1995). Several statistical algorithms have been proposed in the literature for estimating the FDR, the recent and unified procedure based on a nonparametric approach from Strimmer (2008) appearing to be one of the current standard for practitioners. Alternative approaches to FDR estimation consists in viewing the problem as the statistical estimation of the parameters of a finite mixture model. Parametric mixtures for FDR estimation have been considered in, e.g., McLachlan et al. (2006), and semiparametric alternatives have also been proposed (see, e.g., Robin et al., 2007; Chauveau et al., 2014).

For simplicity and easy reproducibility, we only apply here FDR control from the most conservative procedure given by the fdrtool package Strimmer (2008), the local fdr labeled `loc fdr` in the figures. Typical results for the first 4 scaffolds are displayed in Fig. 6. The legend in this Figure also provides the number of cases corresponding to the smallest p-values, that can be rejected at the FDR level $\alpha = 5\%$. This number correspond to the first "time" the curve of the adjusted p-values crosses the horizontal line corresponding to α. Running this procedure on all scaffolds gives the table below, of number of rejected windows per scaffold and treatment, to be compared with number of windows i.e. of multiple tests.

	nb windows	FDR_WD	FDR_WW
Scaf 1	966	430	418
Scaf 2	471	157	143
Scaf 3	403	161	137
Scaf 4	464	199	189
Scaf 5	513	192	166
Scaf 6	538	209	220
Scaf 7	300	120	107
Scaf 8	377	171	171
Scaf 9	259	81	84
Scaf 10	431	162	163
Scaf 11	378	185	173
Scaf 12	298	128	131
Scaf 13	312	155	132
Scaf 14	354	150	137

```
Scaf 15        301   117   109
Scaf 16        283   107    94
Scaf 17        293   158   145
Scaf 18        295   117    99
Scaf 19        320   175   184
```

1.2.3 Mixture-based clustering of means per window

It is also possible to perform a MAP clustering based on the posterior probabilities obtained by fitting a Gaussian mixture model on the sample of window empirical means ($\hat{\mu}_w, w = 1, 2, \ldots$) up to the number of windows for a scaffold, or the total number of windows, for each treatment. This information can be added to the results of the multiple tests procedure and FDR control. Windows leading to significant rejection of H_0 with an empirical window mean $\hat{\mu}_w > \mu_0^t$ should belong to the rightmost component. Fig. 5 gives these EM fits done from the window means for the 19 scaffolds (7556 windows).

1.3 Output results and DMR statistics for FDR level 5%

The final result is a data frame with 7556 rows (one row per window and per scaffold). The columns give Start, number of spots, mean responses and p-values per window, columns for MAP indicators plus a summarizing decision rule in $\{-1, 0, 1\}$ based on the FDR and the sign of the mean shift for each treatment. The decision indicator is:

-1 if the FDR lead to significant case (`reject`) and the sign is negative;

$+1$ if the FDR lead to significant case (`reject`) and the sign is positive;

0 if the FDR concludes to non significant case (`nosig`).

Then the DMR (Differentially Methylated Regions) between the two treatments are computed: these equal `TRUE` only if the two treatments decision rules are different. All these statistics are provided for (and depends upon) the desired FDR level set to 5% here. A summary detailed for each or all scafflod can be also proposed through "Manhattan-like" plots. Fig. 7 displays these Manhattan plots for all the scaffolds.

2 Experiment 2: ECH-SCV-GMN

We follow in this Section the method already presented in Section 1. Thus we just present below the results and specific details.

2.1 Determination of reference means from Gaussian mixtures

We first randomly select 20% of the data as a "reference sample", $n = 47,651$ responses after removal of the 1961 `LOCI` in Scaffold 19. The remaining dataset ($n = 198,448$) is used later for building the multiple test procedure. In view of Fig.8, it is reasonable to assume that the responses for each treatment form an iid sample from a $m = 2$ components univariate Gaussian mixture. For each treatment, the parameters of the model are estimated using the gaussian EM algorithm in the normalmixEM function of the Benaglia et al. (2009) mixtools package. Fig.8 displays the mixture model fits on top of the empirical distributions. The estimated mean of the leftmost, smallest component mean labeled "1" here, is assumed to

be the reference for each treatment "t". Results for the reference means $\hat{\mu}_0^t$ are indicated on Fig.8 and given below together with the corresponding proportions (weights) of the leftmost component:

	SCV	ECH	GMN
component 1 mean	-0.431	-0.214	-0.236
weight (%)	73.2	72.5	74.8

These estimates are computed on the data from all the 19 scaffolds. We have also fit mixture models for each scaffold separately. Fig.9 shows that, for the 3 conditions, the reference means of the leftmost component per scaffold are comparable to the global result.

2.2 Multiple tests on consecutive windows

We assume that the μ_0^t's estimated in Section 1.1 from the 20% reference sample provide reference mean parameters that are now considered non-random. We then build windows of consecutive observations (spots) for the same "nominal" size as before, and test equality of means or distributions between each window and the reference. The windows configuration per scaffold is summarized in Fig. 10.

Fig. 11 illustrates typical responses for treatment, SCV, Scaffold 1, and the first windows $w = 1, \ldots, 10$. This shows that window responses may vary in dispersion or/and in localization, and that the null hypothesis of mean equality may be accepted even though the spots in a window are not close to the reference mean, because of averaging.

2.2.1 Statistical tests per window

As in Experiment 1, it seems not reasonnable to assume Gaussian distributions of responses within each window, so that Student t-tests cannot be used. In addition, about 71% of the windows have fewer than 30 observations here so that asymptotic normality cannot be used either. We use as previously the (non-parametric) Wilcoxon signed rank test of a null hypothesis that the distribution of the sample from each window is symmetric about μ_0^t.

2.2.2 FDR control

We apply as for Experiment 1 the FDR control using the most conservative local fdr procedure given by the fdrtool package Strimmer (2008). Running this procedure on all scaffolds gives the table below, of number of rejected (R) windows per scaffold and treatment, to be compared with number of windows i.e. of multiple tests.

	nb windows	R_SCV	R_ECH	R_GMN
Scaf 1	964	359	256	363
Scaf 2	471	154	92	170
Scaf 3	403	121	92	133
Scaf 4	463	190	123	190
Scaf 5	513	164	105	165
Scaf 6	536	165	128	178
Scaf 7	299	107	92	116
Scaf 8	377	150	109	166
Scaf 9	259	76	43	88
Scaf 10	431	185	96	168
Scaf 11	377	157	132	163
Scaf 12	298	115	102	127

Scaf 13	312	95	76	99
Scaf 14	354	141	112	155
Scaf 15	301	114	67	105
Scaf 16	282	91	77	90
Scaf 17	292	117	88	114
Scaf 18	299	93	67	80
Scaf 19	319	163	157	150

2.2.3 Mixture-based clustering of means per window

We also perform the MAP clustering based on the posterior probabilities obtained by fitting a Gaussian mixture model on the sample of window empirical means ($\hat{\mu}_w, w = 1, 2, \ldots$) up to the total number of windows, for each treatment. Fig. 12 gives these EM fits for the 19 scaffolds (7550 windows).

2.3 Output results and DMR statistics for FDR level 5%

The final result for this experiment is a data frame with 7550 rows (one row per window for each scaffold). The columns give Start, number of spots, mean responses and p-values per window, columns for MAP indicators plus a summarizing decision rule in $\{-1, 0, 1\}$ based on the FDR and the sign of the mean shift for each treatment, already defined in Section 1.3.

Then the DMR (Differentially Methylated Regions) between treatments two-by-two (resp. for the 3 treatments) are computed: these equal `TRUE` only if the two (resp. 3) treatments decision rules are different. All these statistics are provided for (and depend upon) the desired FDR level set to 5% here. Note that for these data only a single case returns a `TRUE` DMR between the 3 treatments: Scaffold 12, window number 50, Start = 1053390, decision rules SVC:0, ECH:1, and GMN:-1.

Fig. 13 gives the "Manhattan-like" plot for scaffold 12, the one for which DMR for the 3 treatments has the single true value. Fig. 14 displays these Manhattan plots for all the scaffolds.

References

Benaglia, T., Chauveau, D., Hunter, D. R., and Young, D. (2009). mixtools: An R package for analyzing finite mixture models. *Journal of Statistical Software*, 32(6):1–29.

Benjamini, Y. and Hochberg, Y. (1995). Controlling the false discovery rate: a practical and powerful approach to multiple testing. *Journal of the Royal Statistical Society, Series B*, 57:289–300.

Bordes, L., Chauveau, D., and Vandekerkhove, P. (2007). A stochastic EM algorithm for a semiparametric mixture model. *Computational Statistics and Data Analysis*, 51(11):5429–5443.

Chauveau, D., Hunter, D. R., and Levine, M. (2015). Semi-parametric estimation for conditional independence multivariate finite mixture models. *Statist. Surv.*, 9:1–31.

Chauveau, D., Saby, N. P. A., Orton, T. G., Lemercier, B., C., W., and Arrouays, D. (2014). Large-scale simultaneous hypothesis testing in monitoring carbon content from french soil database: A semi-parametric mixture approach. *Geoderma*, 219-220:117–124.

McLachlan, G., Bean, R., and Jones, B.-T. (2006). A simple implementation of a normal mixture approach to differential gene expression in multiclass microarrays. *Bioinformatics*, 22:1608–1615.

McLachlan, G. and Peel, D. (2000). *Finite mixture models*. Wiley Series in Probability and Statistics: Applied Probability and Statistics. Wiley-Interscience, New York.

R Core Team (2016). *R: A Language and Environment for Statistical Computing*. R Foundation for Statistical Computing, Vienna, Austria.

Robin, S., Bar-Hen, A., Daudin, J.-J., and Pierre, L. (2007). A semi-parametric approach for mixture models: Application to local false discovery rate estimation. *Computational Statistics and Data Analysis*, 51.

Strimmer, K. (2008). A unified approach to false discovery rate estimation. *BMC Bioinformatics*, 9.

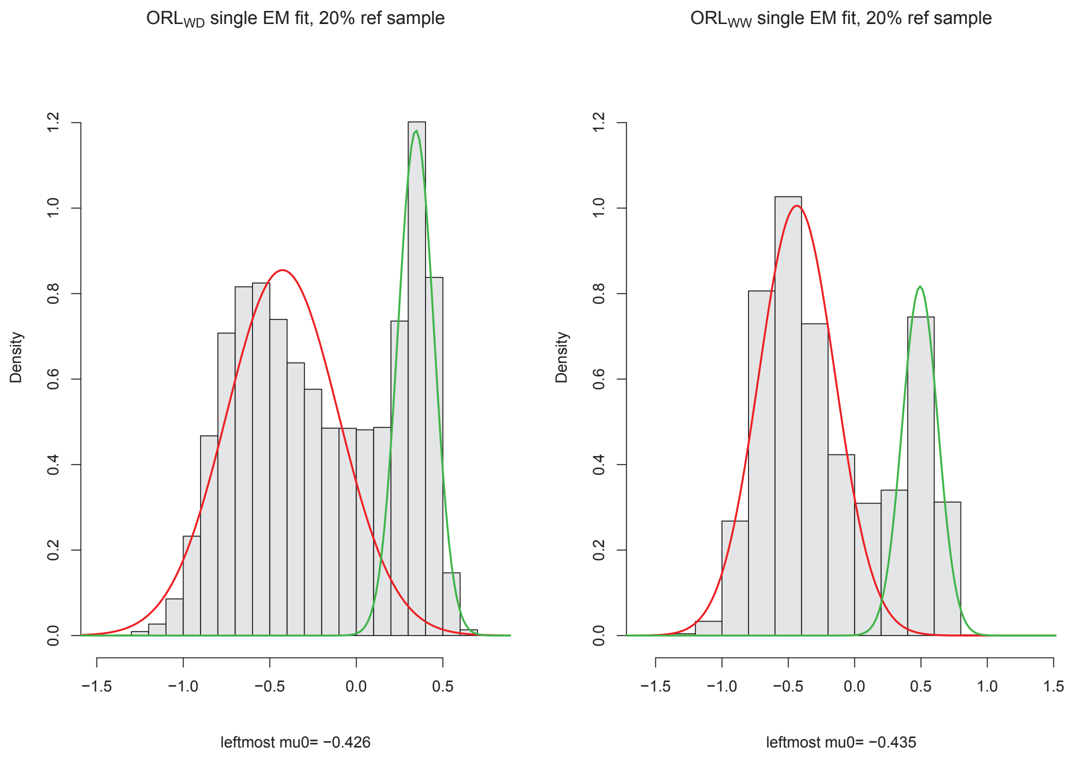

Figure 1: *Experiment 1: EM fits for 2-components Gaussian mixture, for each treatment (ORL_{WD}, ORL_{WW}) considered separately, based on the reference sample (20% of the data). Estimated reference means of leftmost (red) component are indicated.*

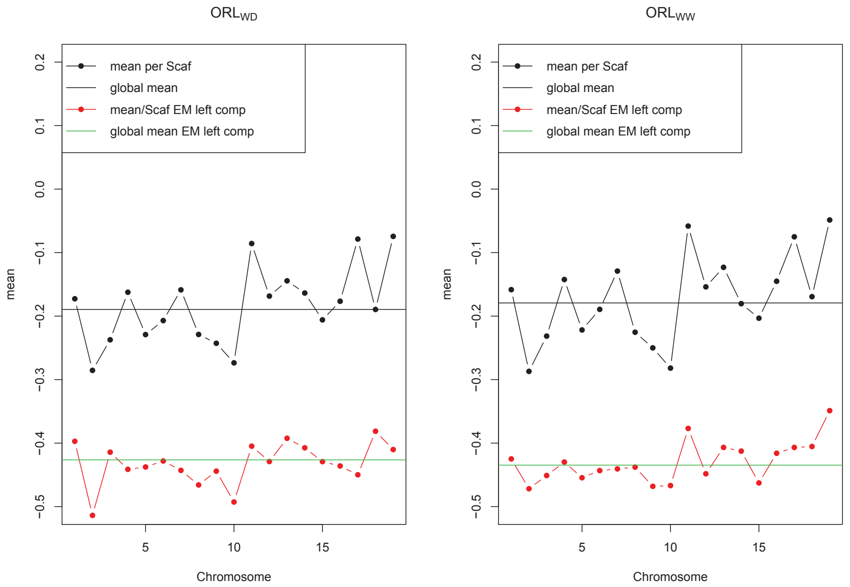

Figure 2: *Experiment 1: EM for 2-components Gaussian mixture single fits for each treatment* (ORL_{WD}, ORL_{WW}) *and per scaffold, based on the reference sample (20% of the data).*

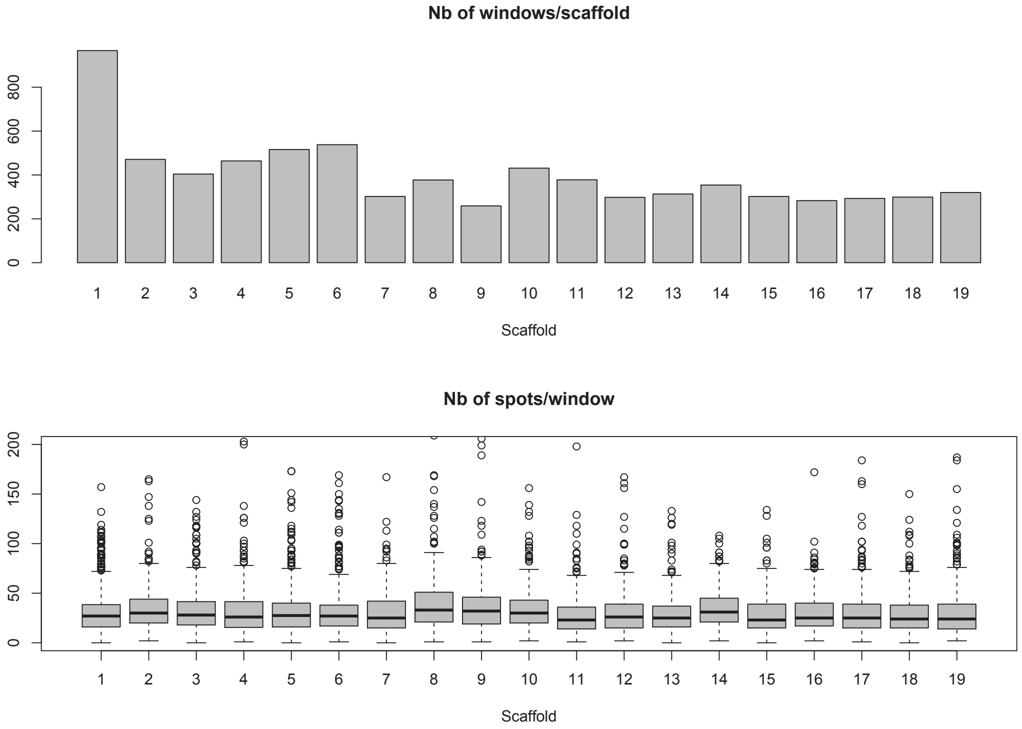

Figure 3: *Experiment 1: Windows configuration for nominal choice 50: Number of windows per scaffold (top); boxplot distributions of the number of spots per window for each scaffold (bottom).*

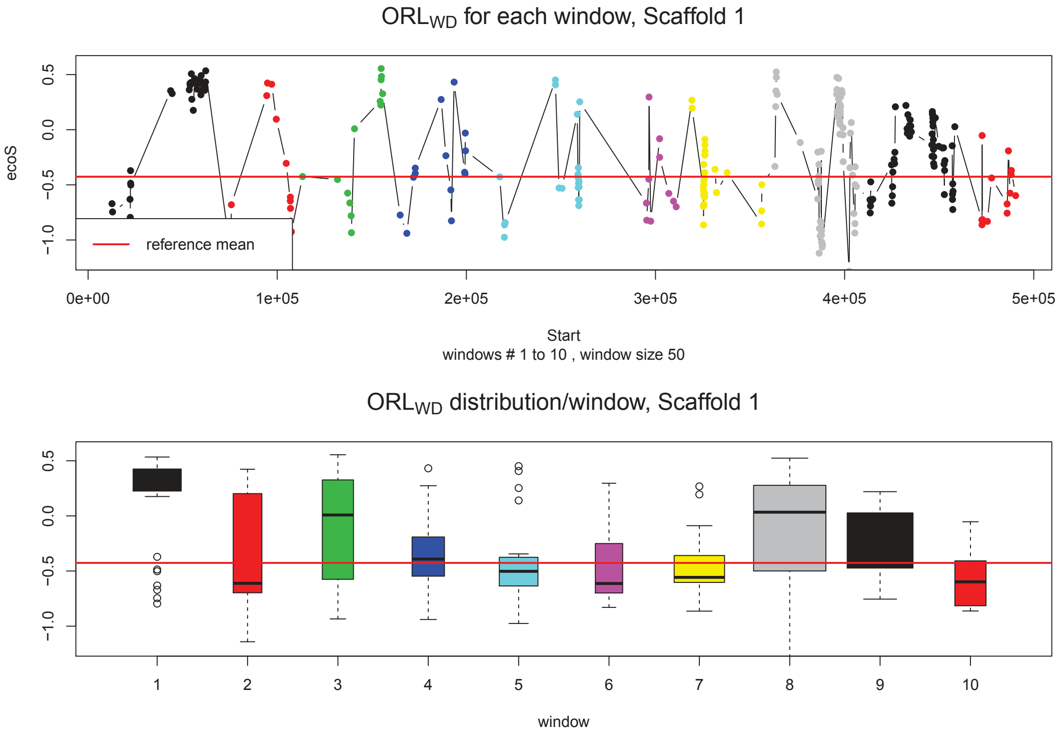

Figure 4: *Experiment 1: Responses ORL_{WD} per window ordered by Start, and boxplot distributions for the 10 first windows, Scaffold 1 (boxplot widths are proportional to number of responses per window). Colors are relative to windows.*

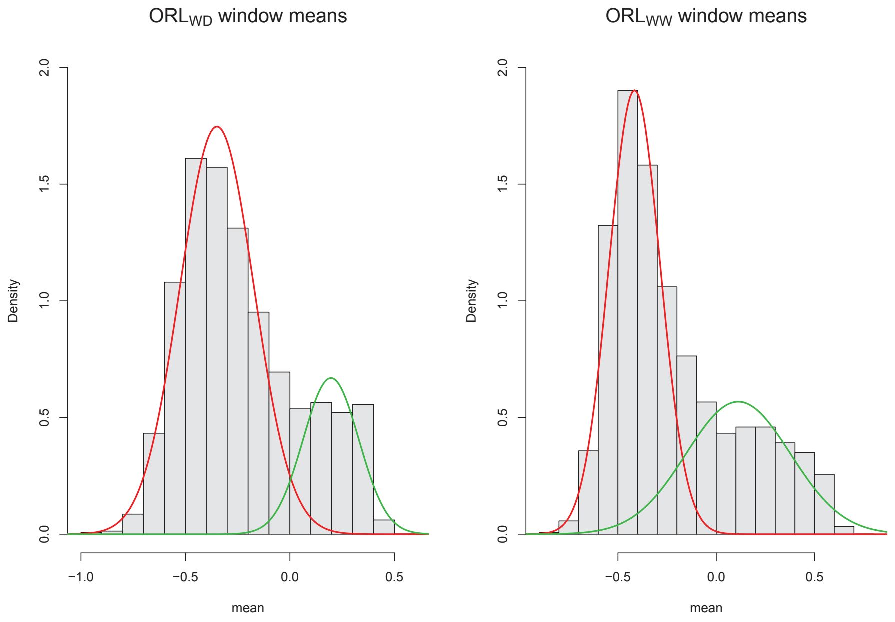

Figure 5: *Experiment 1: Univariate Gaussian EM fits on the mean responses per window, all scaffolds.*

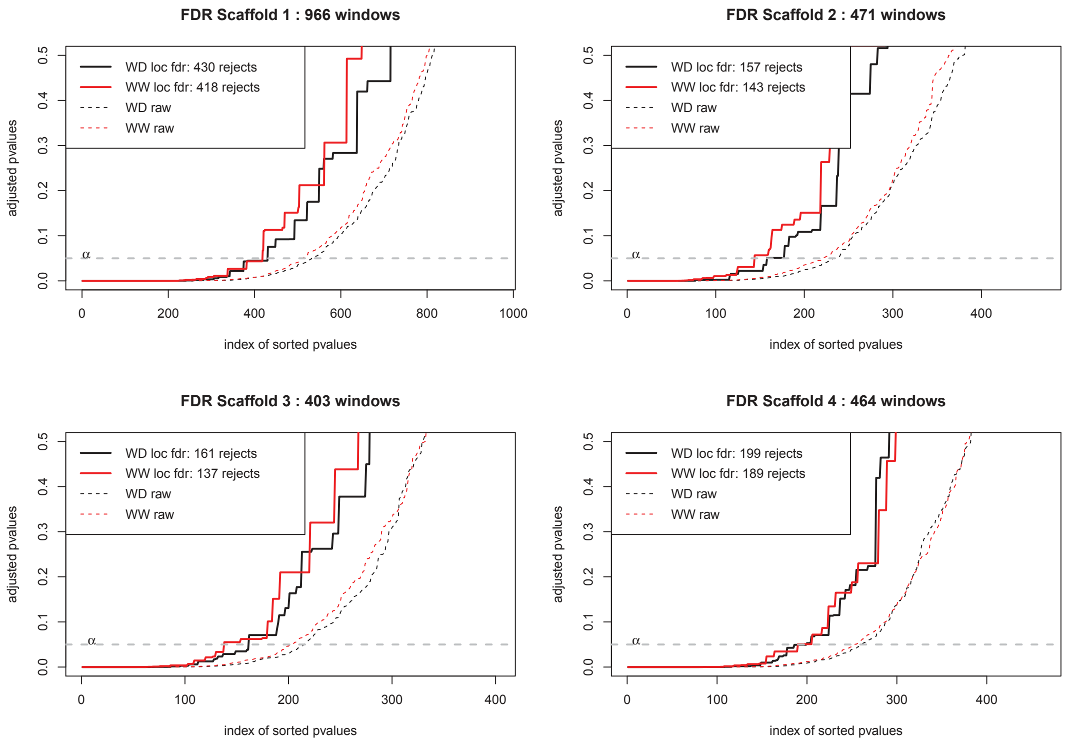

Figure 6: *Experiment 1: FDR control for the first 4 scaffolds. In each case (plot), the FDR control using local fdr, and the raw p-values are provided for the two treatments:* ORL_{WD} *(black),* ORL_{WW} *(red).*

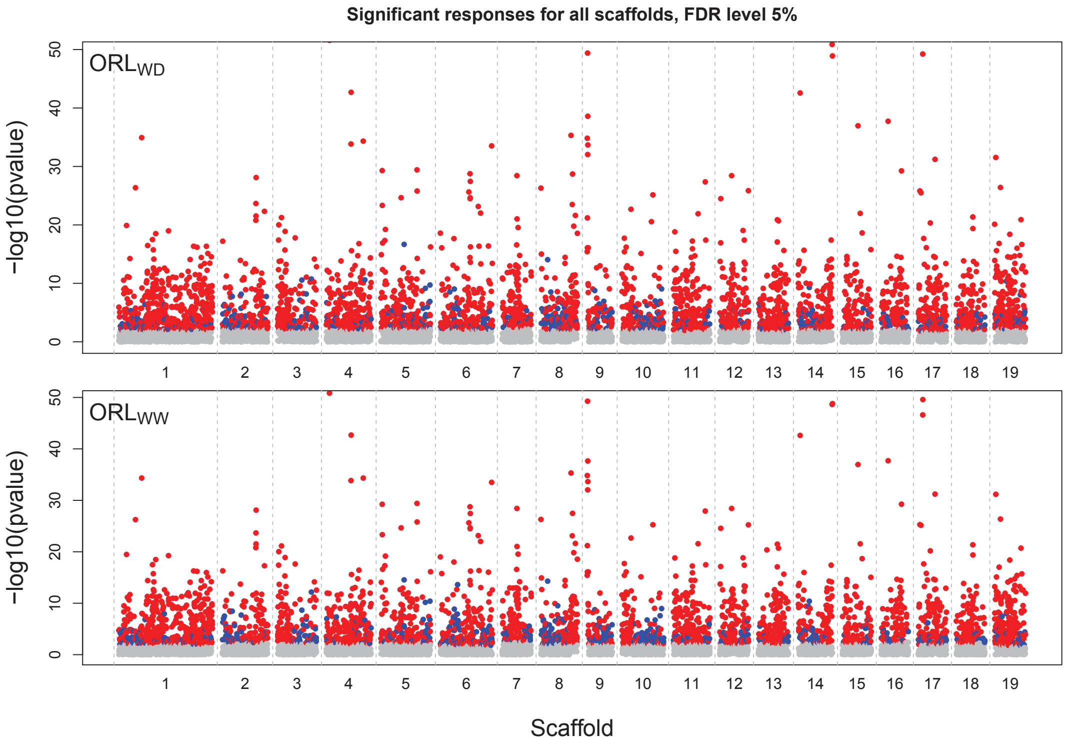

Figure 7: *Experiment 1: Manhattan plots of the p-values for all scaffold, with the color coding of the decision rule* $+1$*(red),* -1*(blue),* 0*(grey).*

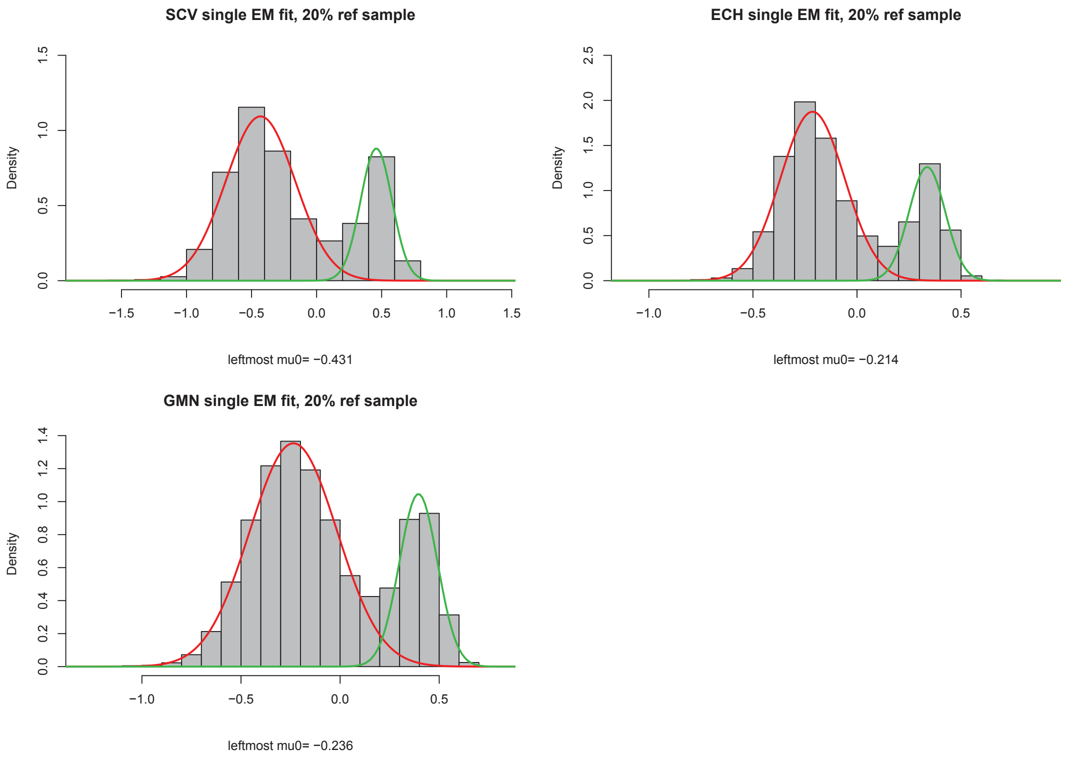

Figure 8: *Experiment 2: EM fits for 2-components Gaussian mixture, for each treatment (SCV, ECH, GMN) considered separately, based on the reference sample (20% of the data). Estimated reference means of leftmost (red) component are indicated.*

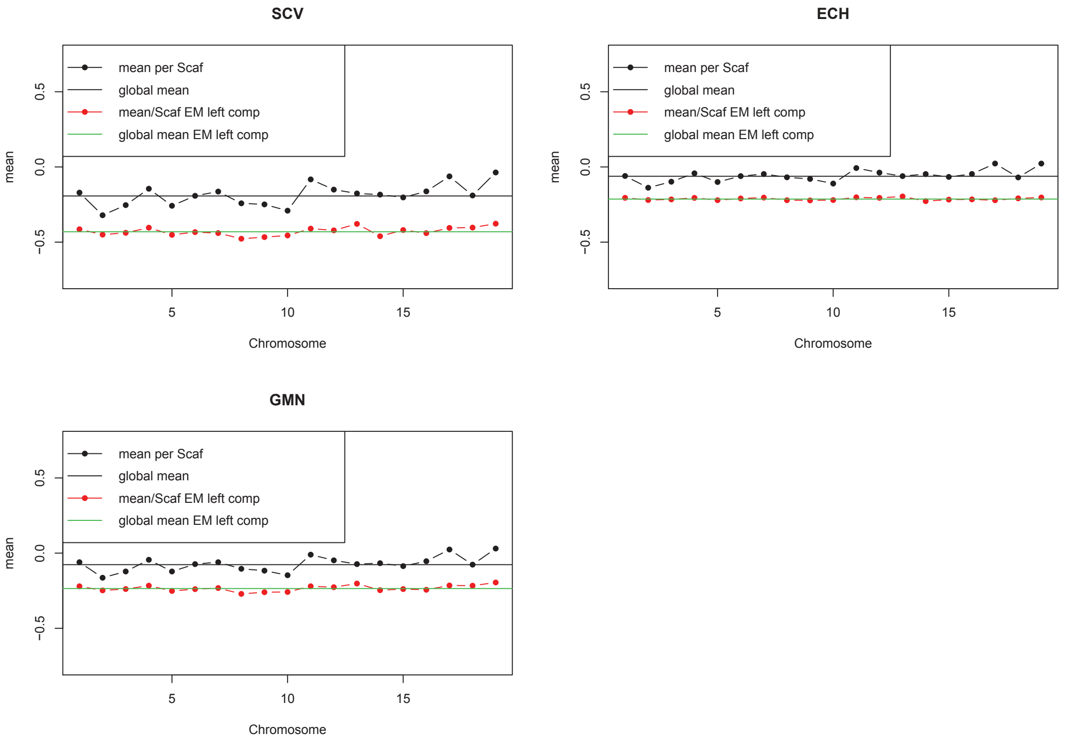

Figure 9: *Experiment 2: EM for 2-components Gaussian mixture single fits for each treatment (SCV, ECH, GMN) and each scaffold, based on the reference sample (20% of the data) without the group* `LOCI` *for scaffold 19.*

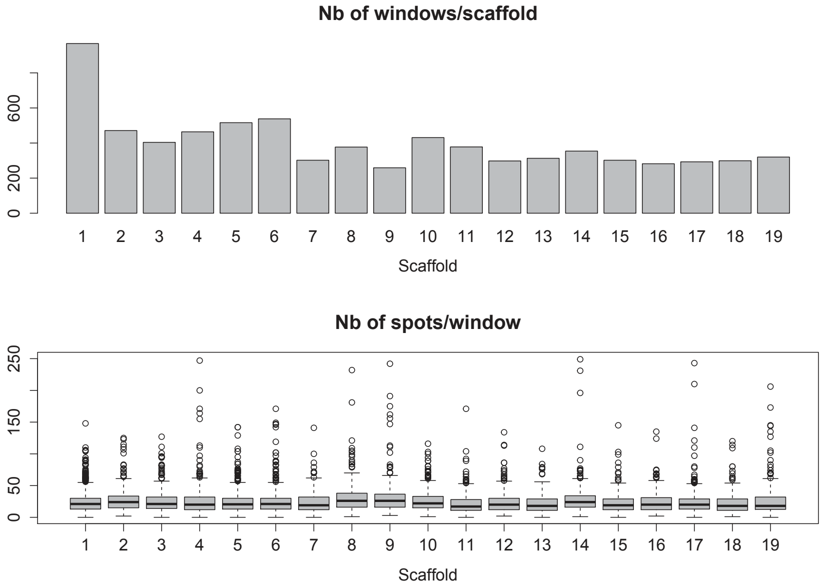

Figure 10: *Experiment 2: Windows configuration for nominal choice 50: Number of windows per scaffold (top); boxplot distributions of the number of spots per window for each scaffold (bottom).*

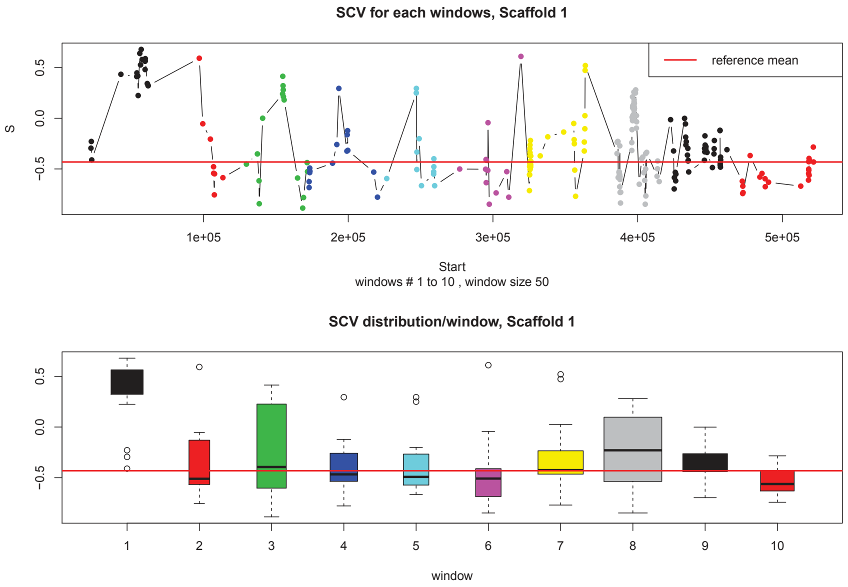

Figure 11: *Experiment 2: Responses SCV per window ordered by Start, and boxplot distributions for the 10 first windows, Scaffold 1 (boxplot widths are proportional to number of responses per window). Colors are relative to windows.*

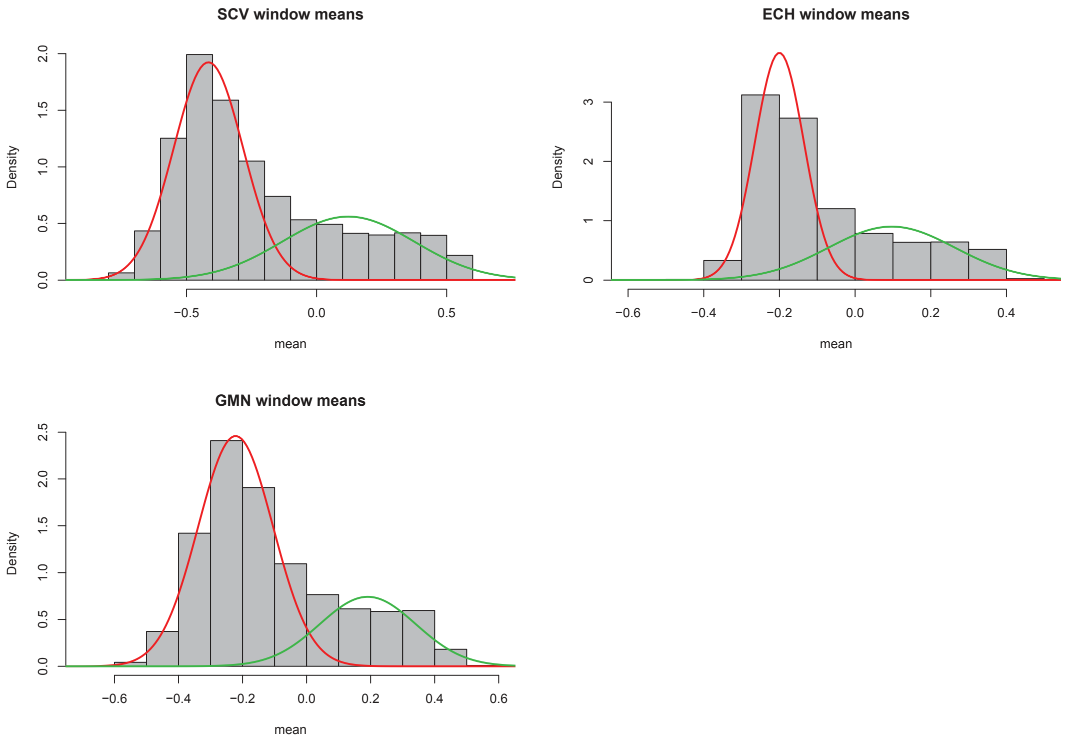

Figure 12: *Experiment 2: Univariate Gaussian EM fits on the mean responses per window, all scaffolds.*

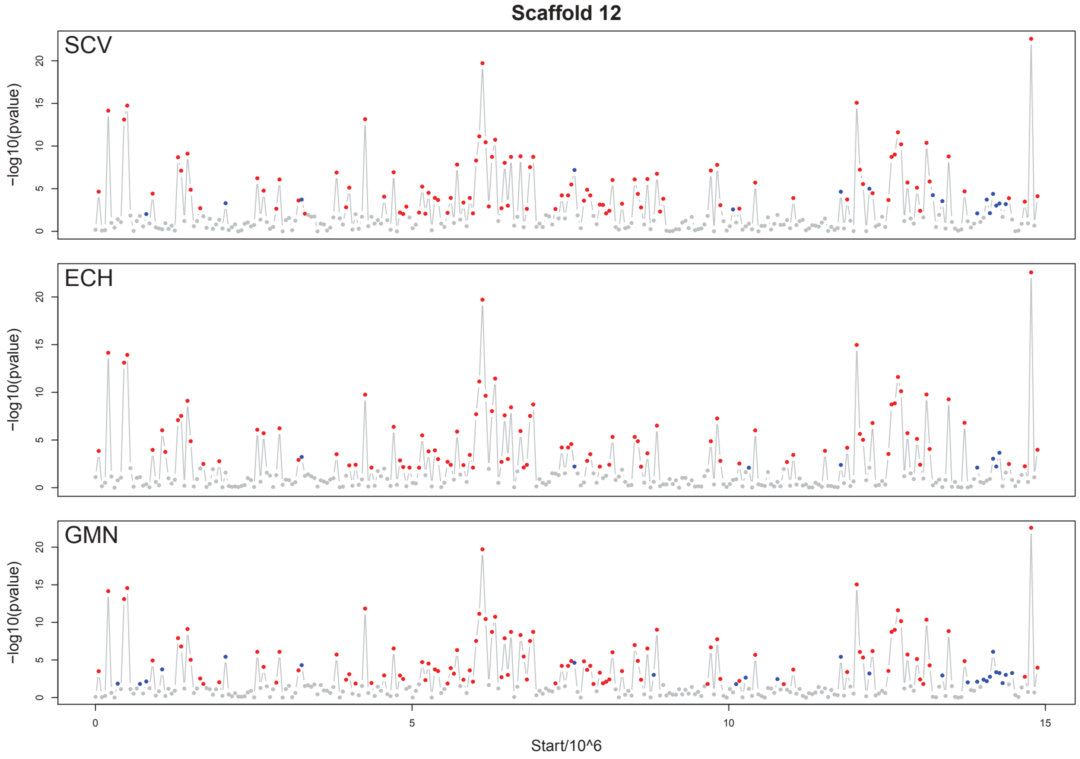

Figure 13: *Experiment 2: Manhattan plots of the p-values for scaffold 12, with the color coding of the decision rule* +1*(red),* −1*(blue),* 0*(grey). This scaffold is the one where DMR for the 3 treatments has a unique true value.*

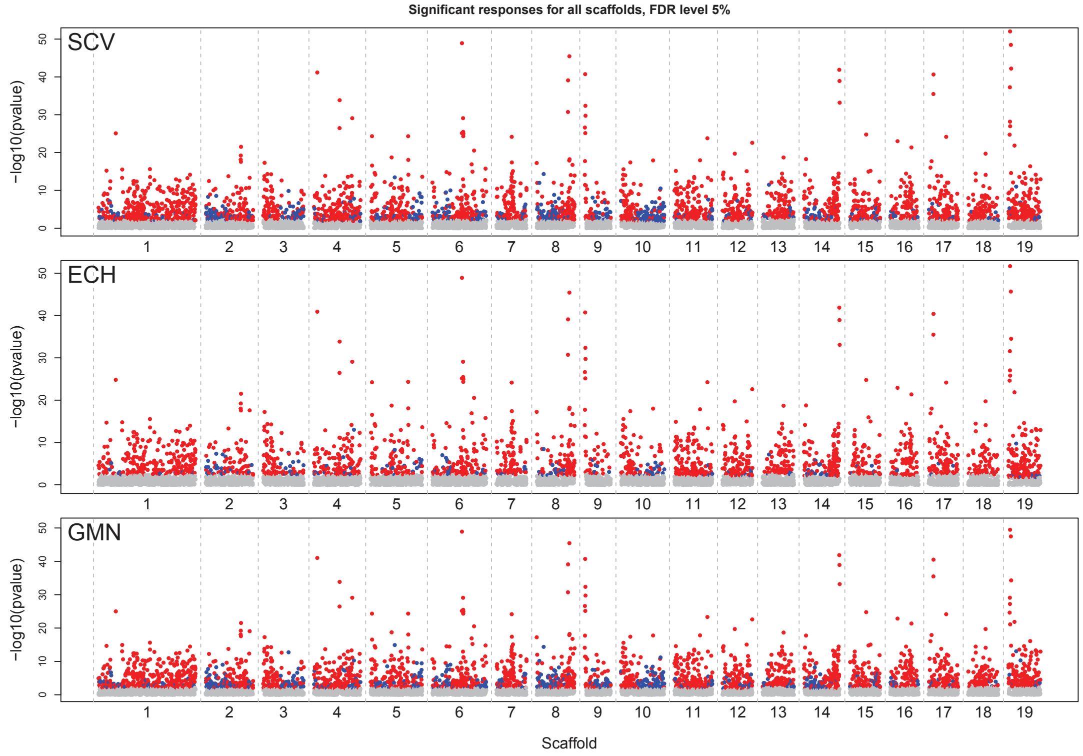

Figure 14: *Experiment 2: Manhattan plots of the p-values for all scaffold, with the color coding of the decision rule* $+1$*(red),* -1*(blue),* 0*(grey).*
